# New Eudesmane-Type Sesquiterpene Glycosides from the Leaves of *Aster koraiensis*

**DOI:** 10.3390/plants9121811

**Published:** 2020-12-21

**Authors:** Ji-Young Kim, Young Hye Seo, Im-Ho Lee, He Yun Choi, Hak Cheol Kwon, Jung-Hye Choi, Jun Lee, Dae Sik Jang

**Affiliations:** 1 Department of Life and Nanopharmaceutical Sciences, Graduate School, Kyung Hee University, Seoul 02447, Korea; k_christina@khu.ac.kr (J.-Y.K.); dlagh93@naver.com (I.-H.L.); choiheyun@khu.ac.kr (H.Y.C.); jchoi@khu.ac.kr (J.-H.C.); 2 Herbal Medicine Resources Research Center, Korea Institute of Oriental Medicine (KIOM), Naju 58245, Korea; wnsl1118@kiom.re.kr; 3 Natural Product Informatics Research Center, Korea Institute of Science and Technology (KIST) Gangneung Institute, Gangneung 25451, Korea; hkwon@kist.re.kr

**Keywords:** *Aster koraiensis*, Compositae, sesquiterpenoids, dicaffeoylquinic acids, nitric oxide, prostaglandin E_2_, anti-inflammation

## Abstract

Four new eudesmane-type sesquiterpenoids, (1*R*,5*S*,6*R*,7*S*,9*S*,10*S*)-1,6,9-trihydroxy-eudesm-3-ene-1,6-di-*O*-*β*-d-glucopyranoside (**1**), (1*R*,5*S*,6S,7*R*,9*S*,10*S*)-1,6,9,11-tetrahydroxy-eudesm-3-ene-1,6-di-*O*-*β*-d-glucopyranoside (**3**), (1*R*,5*S*,6*R*,7*S*,9*S*,10*R*)-9-*O*-(*Z*-*p*-coumaroyl)-1,6,9-trihydroxy-eudesm-3-ene-6-*O*-*β*-d-glucopyranoside (**6**), and (1*R*,5*S*,6*R*,7*S*,9*S*,10*R*)-9-*O*-(*E*-feruloyl)-1,6,9-trihydroxy-eudesm-3-ene-6-*O*-*β*-d-glucopyranoside (**7**), were isolated from a 95% EtOH extract of the leaves of *Aster koraiensis* by repeated chromatography. Moreover, three sesquiterpenoids (**2**, **4**, and **5**) and two caffeoylquinic acids (**8** and **9**) having previously known chemical structures were isolated during the isolation procedure. The four new compounds (**1**, **3**, **6**, and **7**) were elucidated by spectroscopic data (1D- and 2D-NMR, MS, and ECD) interpretation and hydrolysis. Moreover, the absolute configurations of **2**, **4**, and **5** were determined for the first time in this study. The compounds isolated were tested for their viability on nitric oxide (NO) and prostaglandin E_2_ (PGE_2_) production on LPS-stimulated RAW 264.7 cells. Among them, only **7** presented weak inhibitory effects on both NO and PGE_2_ production.

## 1. Introduction

*Aster koraiensis* Nakai (syn. *Gymnaster koraiensis* (Nakai) Kitamura; Compositae) is an endemic species to Korea that is distributed from Jeju Island to the southern parts of Gangwon-do [1]. The young leaves and stems of *A. koraiensis* have been used in Korean cuisines and also used to ornamental plant for beautiful flowers [1]. It has been used for chronic bronchitis, pneumonia, antitussive, and pertussis in Korean folk medicine [2].

It has been reported that the extracts of *A. koraiensis* have a variety of biological activities including anti-proliferative activity on human and mouse tumor cell lines [3,4]. The extracts also exhibited antioxidant and *α*-glucosidase inhibitory activities [5]. Furthermore, it was reported that *A. koraiensis* protects retinal ganglion cells against oxidative stress in diabetic rats [6]. 3,5-*O*-Dicaffeoylepiquinic acid isolated from *A. koraiensis* showed inhibitory activity on AKR1B10 for cancer therapy and on formation of advanced glycation end products (AGEs) to treat diabetic nephropathy [7,8]. Similarly, chlorogenic acid from *A. koraiensis* reduced AGE formation and AGE/RAGE binding activity [9]. A polyacetylene, gymnasterkoreayne B (GKB), from this plant induced a variety of detoxification enzymes and exhibited a hepatoprotective effect and cytotoxicity against cancer cells [10]. Besides, gymnasterkoreayne G, which has a similar chain structure to GKB, has activity on the modulation of NFAT transcription factor [11]. Other polyacetylenes from *A. koraiensis* have acyl CoA: cholesterol acyltransferase (ACAT) activities in rat liver microsomes [12,13].

Previous chemical studies on *A. koraiensis* have led to the identification of several types of secondary metabolites, such as polyacetylenes [3,10,11,12,14], sesquiterpenoids [4,15,16,17], triterpenoids [18], flavonoids [4,16,17], ionones [17], caffeoylquinic acids [16], and benzofurans [16,19]. However, active compounds with anti-inflammatory activities in this plant has been poorly studied. In an ongoing project directed toward the search for bioactive compounds in plants, the leaves of *A. koraiensis* were chosen for phytochemical investigation, since its 95% EtOH extract was found to inhibit production of prostaglandin E_2_ (PGE_2_) and lipopolysaccharide (LPS)-induced nitric oxide (NO) in RAW 264.7 macrophages.

Various chromatographic separation led to the isolation and characterization of four new eudesmane-type sesquiterpenoid glycosides (**1**, **3**, **6**, and **7**) and five known compounds (**2**, **4**, **5**, **8**, and **9**) from the leaves of *A. koraiensis*. The structures of **1**–**7** were elucidated by interpreting one- and two-dimensional (D) nuclear magnetic resonance (NMR) spectroscopic data analysis, enzymatic and acid hydrolysis, and electronic circular dichroism (ECD) calculation. Two caffeoylquinic acids (**8** and **9**) were identified by measurement of NMR spectroscopic data and by comparison with published values. The compounds obtained were evaluated for their activities on the production of the pro-inflammatory mediators, NO and PGE_2_, in RAW264.7 macrophages. We describe in this paper the isolation of compounds from the leaves of *A. koraiensis*, structure elucidation of the seven sesquiterpenes, and inhibitory activities of the isolates against production of NO and PGE_2_.

## 2. Results and Discussion

### 2.1. Structure Elucidation of **1**–**7**

**Figure 1:**
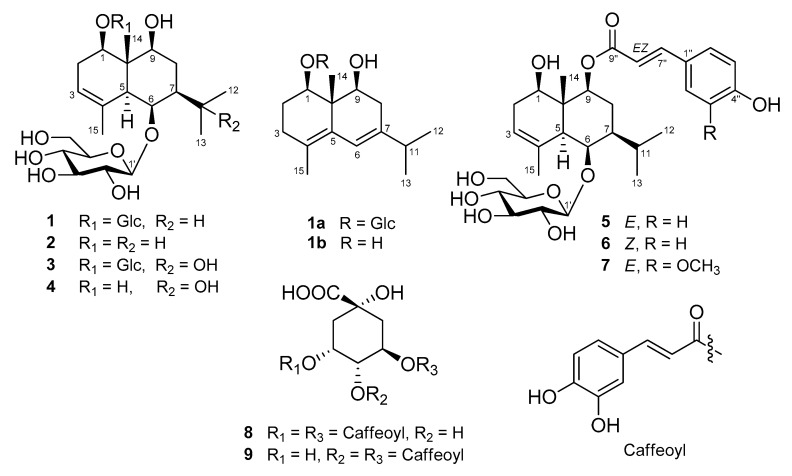
Chemical structures of **1**–**9** isolated from the leaves of *A. koraiensis*.

Four new compounds (**1**, **3**, **6**, and **7**) and five known compounds (**2**, **4**, **5**, **8**, and **9**) were isolated from 95% EtOH extract of the leaves of *A. koraiensis* in the present research (Figure 1).

Compound **1** was isolated as a white powder. The molecular formula of **1** was established as C_27_H_46_O_13_ by HR-ESI-MS (*m/z* = 601.2938 [M + Na]^+^; calcd for C_27_H_46_O_13_Na, 601.2836) (Figure S1). The infrared absorption spectrum showed absorption bands at 3437, 2917, 1358, and 1010 cm^−1^, implicating that **1** has hydroxyl and olefinic groups. The ^1^H-NMR spectroscopic data of **1** exhibited two doublet methyl signals at *δ*_H_ 1.11 (3H, d, *J* = 6.0 Hz) and 0.85 (3H, d, *J* = 6.5 Hz), two singlet methyl signals at *δ*_H_ 2.10 (3H, s) and 1.64 (3H, s), two anomeric protons at *δ*_H_ 4.99 (1H, d, *J* = 8.0 Hz) and 4.92 (1H, d, *J* = 8.0 Hz), and an olefinic proton at *δ*_H_ 5.30 (1H, br s) (Table 1, Figure S2). The ^13^C-NMR spectrum of **1** indicated four methyl carbons (*δ*_C_ 22.0, 21.9 × 2, and 10.7), two methylene carbons (*δ*_C_ 30.3 and 29.4), three oxygenated methine carbons (*δ*_C_ 80.2, 78.4, and 76.6), and four methine carbons (*δ*_C_ 120.4, 52.3, 51.7, and 29.0), 12 carbons (*δ*_C_ 104.6, 102.1, 86.4, 78.4, 78.1, 77.4, 76.2, 75.4, 72.1, 71.6, 63.4, and 62.9) assignable to the glucose moieties including two anomeric carbons and two quaternary carbons (*δ*_C_ 136.7 and 43.0) (Table 1, Figure S3).

**Figure 2:**
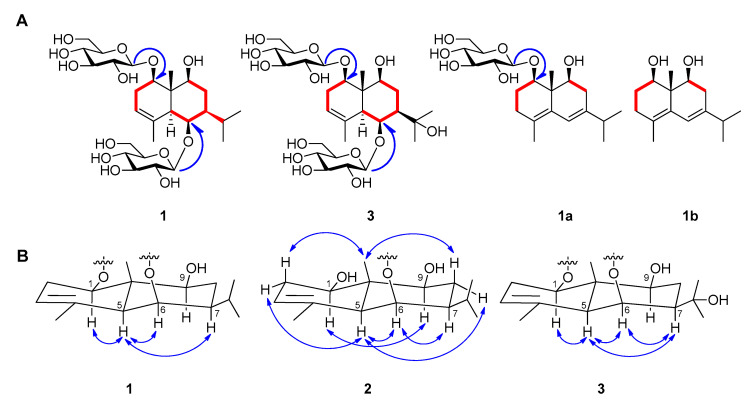
Key COSY (▬) and HMBC (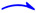) correlations of **1**, **3**, **1a**, and **1b** (**A**). Key NOESY (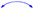) correlations of **1**, **2**, and **3** (**B**).

**Figure 3:**
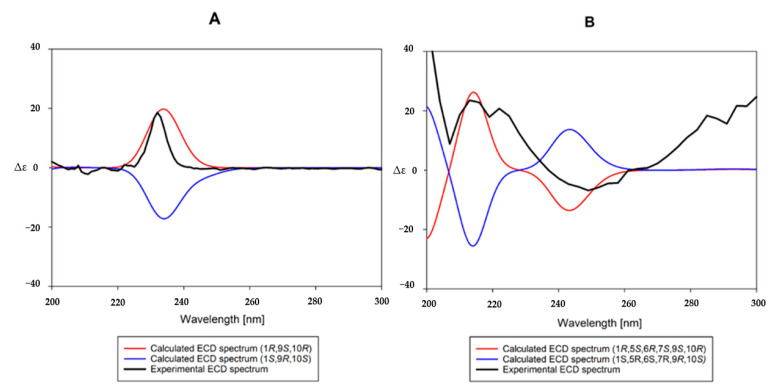
Measured and calculated electronic circular dichroism (ECD) spectra of **1b** (**A**) and **5** (**B**).

Based on the interpretation of ^1^H- and ^13^C-NMR data of **1**, it was inferred that **1** is a eudesmane-type sesquiterpene containing two glucopyranosyl moieties. An enzymatic hydrolysis of **1**, high performance liquid chromatography (HPLC) experiment, and analysis of coupling constants (both 8.0 Hz) of two anomeric protons led to the establishment of the sugars in **1** as *β*-d-glucopyranose. The heteronuclear multiple bond correlation (HMBC) experimental data showed long-range correlations between the signal of H-6 (*δ*_H_ 4.64) and Glc-1’ (*δ*_C_ 104.6) and the signal of H-1 (*δ*_H_ 4.18) and Glc-1” (*δ*_C_ 102.1) (Figure S6), indicating that the positions of the glucopyranosyl moieties are C-1 and C-2. The positions of the three hydroxyl groups were also determined by HMBC correlations to be C-1, 6, and 9. The relative stereochemistry of the hydroxyl groups at C-1, 6, and 9 were determined as all *β*-forms on the basis of the NOESY correlations (H-1 with H-5, H-5 with H-6/H-7) (Figure 2, Figure S7). To make the stereochemistry of **1** clearer, we generated **2** by enzymatic hydrolysis of **1** (Figure 1). The NOESY spectra of **2** revealed correlations of H-1 with H-9, H-5 with H-2*α*/H-6/H-8*α*, H-6 with H-7, and H-14 with H-2*β*/H-8*β* (Figure 2). To determine absolute configurations of **1** and **2**, we obtained a derivative **1a** by acidic hydrolysis of **1** (Figure 1). Additionally, an enzymatic hydrolysis of **1a** was performed to remove the sugar moiety at C-1 and to produce **1b** (Figure 1). In the ^1^H- and ^13^C-NMR spectra of both molecules, there are similar signals that correspond to the protons of the eudesmane-type skeleton, except for the chemical shifts of the proton in C-1 in both **1a** and **1b**, which are different due to the presence or absence of the sugar residue. The absolute configurations at C-1, 9, and 10 of **1b** were established by comparing its experimental ECD spectrum with calculated spectra of (1*R*,9*S*,10*R*) and (1*S*,9*R*,10*S*) models using the time-dependent density functional theory (TDDFT) method. The experimental ECD spectrum of **1b** displayed a positive Cotton effect (CE) at 232 nm (∆ε + 24.8). The experimental data were in good agreement with the calculated ECD spectrum of the (1*R*,9*S*,10*R*) model (Figure 3), suggesting the absolute configuration of **1b** as (1*R*,9*S*,10*R*). Therefore, the structure of the new compound **1** was elucidated as (1*R*,5*S*,6*R*,7*S*,9*S*,10*S*)-1,6,9-trihydroxy-eudesm-3-ene-1,6-di-*O*-*β*-d-glucopyranoside.

The planar structure of **2** turned out to be the same as 1*β*,6*β*,9*α* -trihydroxy-*trans*-eudesm-3-ene-6-*O*-*β*-d-glucopyranoside, which was isolated previously from the flower of *A. koraiensis* and reported only its relative configuration [4]. Although their ^1^H- and ^13^C-NMR spectroscopic data were identical, indicating they are the same compounds, we found that **2** has different configuration (9*β*-hydroxy-) from the other one (9*α*-hydroxy-). Thus, we propose the structure of the known compound **2** as (1*R*,5*S*,6*R*,7*S*,9*S*,10*R*)-1,6,9-trihydroxy-eudesm-3-ene-6-*O*-*β*-d-glucopyranoside.

Compound **3** was isolated as a white powder. The molecular formula of **3** was established as C_27_H_46_O_14_ by HR-ESI-MS (*m*/*z* = 617.2791 [M + Na]^+^; calcd for C_27_H_46_O_14_Na, 617.2785) (Figure S15). The ^1^H-NMR spectrum of **3** exhibited two singlet methyl signals at *δ*_H_ 1.58 (3H) and 1.38 (3H), two anomeric protons at *δ*_H_ 4.99 (1H, d, *J* = 8.0 Hz) and 4.87 (1H, d, *J* = 8.0 Hz), and an olefinic proton at *δ*_H_ 4.98 (1H, br s) (Table 1, Figure S16). The ^13^C-NMR spectrum of **3** showed 27 signals including four methyl carbons (*δ*_C_ 29.5, 29.2, 22.4, and 10.9), two methylene carbons (*δ*_C_ 28.5 and 27.3), 12 signals for glucose moieties, and three quaternary carbons (*δ*_C_ 136.7, 72.6, and 42.9) (Table 1, Figure S17). The NMR data for **3** were very similar to **1** except for the presence of a quaternary oxygenated carbon signal instead of a methine carbon signal. The position of the quaternary carbon at C–11 was deduced on the basis of the coupling pattern for two methyl groups changed from (*δ*_H_ 1.11 (3H, d, *J* = 6.0 Hz) and 0.85 (3H, d, *J* = 6.5 Hz)) to (*δ*_H_ 1.58 (3H, s) and 1.38 (3H, s)) in the ^1^H-NMR spectrum. It was supported by HMBC correlations between the signal of C-11 and H-12, H-13, and H-7 (Figure S20). The relative stereochemistry of hydroxyl groups at C-1, 6, and 9 were determined as all *β*-forms like **1** by analyzing NOESY correlations (H-1 with H-5, H-5 with H-6/H-7, and H-6 with H-7), indicating **3** is a 11-hydroxy derivative of **1** (Figure 2, Figure S21). Considering a biogenetic relationship with **1**, the structure of the new compound **3** was proposed as (1*R*,5*S*,6*S*,7*R*,9*S*,10*S*)-1,6,9,11-tetrahydroxy-eudesm-3-ene-1,6-di-*O*-*β*-d-glucopyranoside.

The planar structure of **4** was also reported from the flowers of *A. koraiensis* together with **2** (Figure 2) [4]. The authors reported the chemical structure of the compound as 1*β*,6*β*,9*α*,11-tetrahydroxy-*trans*-eudesm-3-ene-6-*O*-*β*-d-glucopyranoside on the basis of the NOESY experiment. The ^1^H- and ^13^C-NMR spectroscopic data of **4** were identical with those of published values [4]. However, the NOESY correlations indicated that the relative configuration of **4** is the same as **1**–**3**. Therefore, we propose the structure of the known compound **4** as (1*R*,5*S*,6*S*,7*R*,9*S*,10*R*)-1,6,9,11-tetrahydroxy-eudesm-3-ene-6-*O*-*β*-d-glucopyranoside.

**Figure 4:**
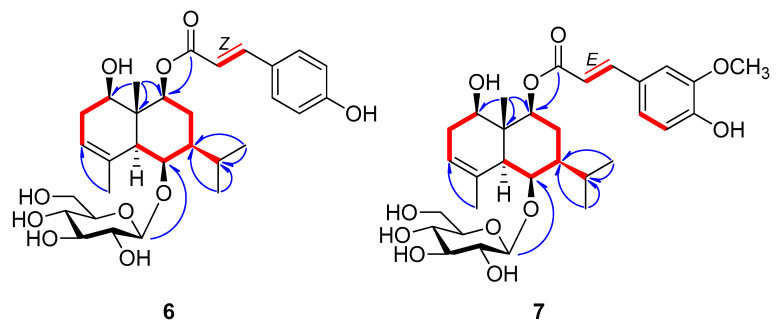
Key ^1^H-^1^H COSY (▬) and HMBC (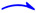) correlations of **6** and **7**.

Compound **6** was obtained as a pale yellow amorphous powder and the molecular formula was established as C_30_H_42_O_10_ by HR–ESI–MS (*m*/*z* 561.2710 [M-H]^−^, calcd for C_30_H_41_O_10_, 561.2705) (Figure S15). The ^1^H-NMR spectrum of **6** showed four methyl signals at *δ*_H_ 1.88 (3H, s), 1.26 (3H, s), 1.02 (3H, d, *J* = 6.5 Hz), and 0.93 (3H, d, *J* = 6.5 Hz); one anomeric proton at *δ*_H_ 4.38 (1H, d, *J* = 8.0 Hz; and *cis*-olefinic group at *δ*_H_ 6.89 (1H, d, *J* = 12.5 Hz) and 5.80 (1H, d, *J* = 12.5 Hz) (Table 2, Figure S16). The ^13^C-NMR spectrum of **6** exhibited four methyl signals (*δ*_C_ 22.0, 21.9, 21.8, and 11.8), six glucosyl signals including an anomeric carbon (*δ*_C_ 104.5, 78.5, 77.5, 76.2, 72.1, and 63.3), three oxymethine signals (*δ*_C_ 80.8, 80.3, and 76.0), and *cis*-*para*-coumaroyl group (*δ*_C_ 167.8, 160.3, 145.7, 134.2 × 2, 127.9, 117.7, and 116.0 × 2), indicating that **6** is a *p*-coumaroyl derivative of **2** (Table 2, Figure S17). The positions of hydroxyl, *p*-coumaroyl and *β*-d-glucopyranosyl groups and the relative configurations were determined by analysis of the HMBC and NOESY correlations (Figure 4, Figures S20 and S21). On the basis of the NMR data and a biogenetic relationship with **1**-**4**, the structure of the new compound **6** was proposed as (1*R*,5*S*,6*R*,7*S*,9*S*,10*R*)-9-*O*-(*Z*-*p*-coumaroyl)-1,6,9-trihydroxy-eudesm-3-en-6-*O*-*β*-d-glucopyranoside. A literature survey revealed that **6** is the geometric isomer of **5**, 9*β*-*O*-(*E*-*p*-hydroxycinnamoyl)-1*β*,6*β*-dihydroxy-*trans*-eudesm-3-ene-6-*O*-*β*-d-glucopyranoside, which was isolated previously from the aerial parts of *A. koraiensis* and reported only its relative configuration [16]. The absolute configuration of **5** was established by comparing its experimental ECD spectrum with those calculated spectra of (1*R*,5*S*,6*R*,7*S*,9*S*,10*R*) and (1*S*,5*R*,6*S*,7*R*,9*R*,10*S*) models using the same method as **1c**. The experimental data (Figure 3) were in accordance with the calculated ECD spectrum of the (1*R*,5*S*,6*R*,7*S*,9*S*,10*R*) model, offering the absolute configuration of **5** as (1*R*,5*S*,6*R*,7*S*,9*S*,10*R*)-9-*O*-(*E*-*p*-coumaroyl)-1,6,9-trihydroxy-eudesm-3-ene-6-*O*-*β*-d-glucopyranoside and also supporting our proposed absolute configurations for **1**–**6**.

The molecular formula of **7** was established as C_31_H_44_O_11_ by HR-ESI-MS (*m*/*z* 575.2848 [M-H_2_O-H]^−^, calcd for C_31_H_43_O_10_, 575.2856) (Figure S22). The ^1^H- and ^13^C-NMR spectroscopic data of **7** were very similar with those of **5** and **6** except for the presence of *E*-*p*-feruloyl group in **7** instead of *E*- or *Z*-*p*-coumaroyl group (Table 2). An ABX system (*δ*_H_ 6.81 (d, *J* = 8.5 Hz), 7.08 (dd, *J* = 8.5, 2.0 Hz), and 7.22 (d, *J* = 2.0 Hz)), *trans*-olefinic group (*δ*_H_ 7.61 (1H, d, *J* = 16.0 Hz) and 6.38 (1H, d, *J* = 16.0 Hz)), and a methoxy signal (*δ*_H_ 3.91 (3H, s)) were revealed in the ^1^H-NMR spectrum of **7**, indicating the presence of *E*-*p*-feruloyl group in **7**. The positions of the functional groups in **7** and the relative configuration were confirmed by the HMBC and NOESY correlations (Figure 4, Figures S26 and S27). Thus, from the analysis of above data, the structure of the new compound **7** was elucidated as (1*R*,5*S*,6*R*,7*S*,9*S*,10*R*)-9-*O*-(*E*-*p*-feruloyl)-1,6,9-trihydroxy-eudesm-3-ene-6-*O*-*β*-d-glucopyranoside.

The structures of other known compounds were identified as 3,5-dicaffeoylquinic acid (**8**) [20] and 4,5-dicaffeoylquinic acid (**9**) [20] by comparison of their NMR data with those reported.

Although numerous types of sesquiterpene have been reported from the Compositae family, until now, only eudesmane-type sesquiterpenes have been isolated from *A. koraiensis* [15,16,17], except for the presence of an oplopane-type sesquiterpene [15].

### 2.2. Inhibitory Activities of the Isolates on LPS-Stimulated NO and PGE_2_ Production

**Figure 5:**
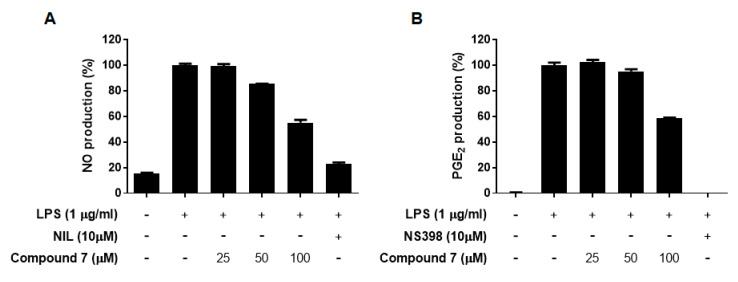
Inhibitory effects of compound **7** on LPS-stimulated NO (**A**) and PGE_2_ productions (**B**) in RAW 264.7 macrophages. Cells were pretreated with different concentrations (25, 50, or 100 μM) of compound **7** for 1 h, then with LPS (1 μg/mL), and then were incubated for 24 h. l-*N*^6^-(1-Iminoethyl)lysine (l-NIL, 10 μM) and *N*-[2-(cyclohexyloxy)-4-nitrophenyl]-methanesulfonamide (NS-398, 10 μM) were used as positive NO and PGE_2_ production inhibitors, respectively.

All the isolates **1**–**9** obtained from the leaves of *A. koraiensis* were evaluated for their inhibitory effects of LPS-stimulated NO and PGE_2_ production in RAW 264.7 macrophages at non-toxic concentrations (Table 3). Of these, only the new compound **7** presented weak inhibitory effects on both NO and PGE_2_ production with observed IC_50_ values of 95.7 and 111.6 μM, respectively, while others were inactive (Table 3, Figure 5).

## 3. Materials and Methods

### 3.1. Plant Material

The leaves of *Aster koraiensis* Nakai (Compositae) were collected at Pyeongchang, Gangwon-Do, Korea, in 2017. The origin of the plant was authenticated by one of the authors D.S.J. and a voucher specimen (ASKO1-2017) was deposited at the College of Pharmacy, Kyung Hee University, Korea.

### 3.2. General Experimental Procedures

General experimental procedures are in the Supplementary Materials.

### 3.3. Extraction and Isolation

The dried and ground leaves (5.0 kg) of *A. koraiensis* were extracted twice with 25 L of 95% EtOH at 70 °C for 3 hours and extract solutions were condensed using a steam heated evaporator. The 95% EtOH extract (500 g) was chromatographed over Diaion HP-20 (9.8 × 63.0 cm) eluting with an acetone-H_2_O gradient (from 0:1 to 1:0 *v/v*) to afford 28 fractions (C1 ~ C28).

Fraction C4 (11.82 g) was separated into five subfractions by Sephadex LH-20 column chromatography (CC) (4.8 × 63.0 cm) with 50 % acetone (C4-1 ~ C4-5). Compound **3** (18.7 mg) was purified by repetitive chromatography from subfraction C4-2 (2.99 g). Fraction C5 (8.32 g) was fractionated further by Sephadex LH-20 CC (4.8 × 43.0 cm) with MeOH-H_2_O (1:1 *v/v*), yielding 14 fractions (C5-1 ~ C5-14) and compound **1** (1.76 g). Compound **4** (61.3 mg) was purified using a flash chromatography system (Redi Sep-C18 cartridge, 43 g, MeOH-H_2_O gradient (from 10:90 to 40:60 *v/v*)) from fraction C5-3-5 (133.8 mg). Compound **1** (324.1 mg) was additionally obtained by flash chromatography with Redi Sep-C18 cartridge (120 g, MeOH-H_2_O, from 20:80 to 50:50 *v/v*) from fraction C5-3-11 (701.6 mg). Fraction C9 (3.64 g) was subjected to Sephadex LH-20 CC with a MeOH-H_2_O mixture (1:1 *v/v*) to give nine subfractions (C9-1 ~ C9-9). Subfraction C9-5 (522.9 mg) was separated further using a flash chromatography system with Redi Sep-C18 (43 g, MeOH-H_2_O, 40:60 to 70:30, *v/v*) to afford compound **2** (41.8 mg). Fraction C11 (10.0 g) was separated into seven subfractions (C11-1 ~ C11-7) by Sephadex LH-20 CC (3.6 × 65.0 cm) with 50% acetone. Subfraction C11-6 (2.27 g) was chromatographed over silica gel (230–400 mesh; 4.8 × 28.3 cm) with an EtOAc-acetone-H_2_O mixture (from 50:45:5 to 30:60:10 *v/v/v*) as mobile phase to obtain compounds **8** (948.8 mg) and **9** (10.2 mg). Fraction C17 (3.12 g) was fractionated into five subfractions (C17-1 ~ C17-5) by Sephadex LH-20 CC (4.0 × 69.0 cm) with MeOH-H_2_O (1:1 *v/v*). Compounds **5** (25.6 mg), **6** (7.6 mg), and **7** (4.8 mg) were isolated from subfraction C17-3 (300.0 mg) using a flash chromatography system with silica cartridge (48 g, CH_2_Cl_2_-MeOH-H_2_O, 35:65 to 50:50, *v/v*).

#### 3.3.1. (1*R*,5*S*,6*R*,7*S*,9*S*,10*S*)-1,6,9-trihydroxy-eudesm-3-ene-1,6-di-*O*-*β*-d-glucopyranoside (**1**)

White powder; m.p.: 167.2 °C; ${\left[\alpha \right]}_{D}^{20}$: −0.4° (*c* 0.1, MeOH); UV (acetonitrile) λ_max_ (log ε): 205 (3.83), 376 (4.08) nm; IR (ATR) ν_max_ 3437, 2917, 1358, 1010 cm^−1^; HR-ESI-MS *m*/*z* = 601.2838 [M + Na]^+^, (calcd for C_27_H_46_O_13_Na, 601.2836); NMR data: Table 1.

#### 3.3.2. 1*R*,9*S*,10*S*-1,9-Dihydroxy-eudesm-4,6-diene-1-*O*-*β*-d-glucopyranoside (**1a**)

Pale yellow amorphous powder; HR-Q-TOF-MS *m/z* = 397.2237 [M–H]^−^ (calcd for C_21_H_33_O_7_, 397.2226); ${\left[\alpha \right]}_{D}^{20}$: 58.3° (*c* 0.03, MeOH); UV (MeOH) λ_max_ nm (log ε): 240 (3.37), 296 (3.34); IR (ATR) ν_max_ 1937, 1587, 1348, 1013 cm^−1^; ^1^H-NMR (500 MHz, CD_3_OD) *δ*_H_ 6.05 (1H, brs, H-6), 4.48 (1H, d, *J* = 8.0 Hz, Glc-1), 3.94 (1H, dd, *J* = 12.0, 4.0 Hz, H-1), 3.88 (1H, dd, *J* = 12.0, 2.0 Hz, Glc-6a), 3.83 (1H, dd, *J* = 11.0, 5.5 Hz, H-9), 3.65 (1H, dd, *J* = 12.0, 6.0 Hz, Glc-6b), 3.36 (1H, m, Glc-5), 3.29 (1H, m, Glc-4), 3.27 (1H, m, Glc-3), 3.17 (1H, dd, *J* = 9.5, 8.0 Hz, Glc-2), 2.31 (1H, m, H-11), 2.19 (2H, m, H-8), 2.08 (2H, m, H-3), 1.93 (2H, m, H-2), 1.69 (3H, s, H-15), 1.07 (3H, d, *J* = 2.0 Hz, H-12), 1.06 (3H, d, *J* = 2.0 Hz, H-13), 1.01 (3H, s, H-14); ^13^C–NMR (125 MHz, CD_3_OD) *δ*_C_ 141.9 (C-7), 133.3 (C-5), 129.1 (C-4), 118.5 (C-6), 102.4 (Glc-1), 84.9 (C-1), 78.6 (Glc-5), 78.3 (Glc-3), 78.1 (C-9), 75.1 (Glc-2), 71.7 (Glc-4), 63.0 (Glc-6), 43.1 (C-10), 36.4 (C-11), 33.0 (C-8), 32.5 (C-3), 24.6 (C-2), 22.2 (C-12), 21.7 (C-13), 19.2 (C-15), 12.5 (C-14).

#### 3.3.3. 1*R*,9*S*,10*R*-1,9-Dihydroxy-eudesm-4,6-diene (**1b**)

Pale yellow amorphous powder; ${\left[\alpha \right]}_{D}^{20}$: 67.2° (*c* 0.03, MeOH); UV (MeOH) λ_max_ nm (log ε): 239 (3.27), 290 (3.22); IR (ATR) ν_max_ 1569, 1418, 1363, 1016 cm^−1^; ^1^H–NMR (500 MHz, CD_3_OD) *δ*_H_ 6.07 (1H, brs, H-6), 3.77 (1H, dd, *J* = 12.0, 4.0 Hz, H-1), 3.68 (1H, dd, *J* = 11.0, 5.5 Hz, H-9), 2.30 (1H, m, H-11), 2.22 (2H, m, H-8), 2.14 (2H, m, H-3), 1.80 (2H, m, H-2), 1.68 (3H, s, H-15), 1.07 (3H, d, *J* = 2.0 Hz, H-12), 1.05 (3H, d, *J* = 2.0 Hz, H-13), 0.93 (3H, s, H-14); ^13^C–NMR (125 MHz, CD_3_OD) *δ*_C_ 141.9 (C-7), 133.3 (C-5), 129.1 (C-4), 118.5 (C-6), 78.6 (C-1), 78.3 (C-9), 43.1 (C-10), 36.4 (C-11), 33.0 (C-8), 32.5 (C-3), 24.6 (C-2), 22.2 (C-12), 21.7 (C-13), 19.2 (C-15), 12.5 (C-14).

#### 3.3.4. (1*R*,5*S*,6*S*,7*R*,9*S*,10*S*)-1,6,9,11-tetrahydroxy-eudesm-3-ene-1,6-di-*O*-β-d-glucopyranoside (**3**)

White powder; m.p.: 230 °C; ${\left[\alpha \right]}_{D}^{20}$: −1.5° (*c* 0.1, MeOH); UV (acetonitrile) λ_max_ (log ε): 205 (3.85), 322 (4.02), 359 (4.08), 373 (4.43) nm; IR (ATR) ν_max_ 3314, 2876, 1360, 1011 cm^−1^; HR-ESI-MS (positive mode) *m*/*z* = 617.2791 [M + Na]^+^ (calcd for C_27_H_46_O_14_Na, 617.2785); NMR data: Table 1.

#### 3.3.5. (1*R*,5*S*,6*R*,7*S*,9*S*,10*R*)-*O*-(*Z*-*p*-coumaroyl)-1,6,9-Trihydroxy-eudesm-3-ene-6-*O*-β-d-glucopyranoside (**6**)

Pale yellow amorphous powder; HR-ESI-MS *m/z* = 561.2701 [M–H]^−^ (calcd for C_30_H_41_O_10_, 561.2705); ${\left[\alpha \right]}_{D}^{20}$: 48.4° (*c* 0.1, MeOH); UV (MeOH) λ_max_ nm (log ε): 313 (4.48); IR (ATR) ν_max_ 1705, 1603 1513, 1165, 1074, 989 cm^−1^; NMR data: Table 2.

#### 3.3.6. (1*R*,5*S*,6*R*,7*S*,9*S*,10*R*)-*O*-(*E*-feruloyl)-1,6,9-Trihydroxy-eudesm-3-ene-6-*O*-β-d-glucopyranoside (**7**)

Pale yellow amorphous powder; HR-ESI-MS *m/z* = 575.2845 [M–H_2_O–H]^−^ (calcd for C_31_H_43_O_10_, 575.2856); ${\left[\alpha \right]}_{D}^{20}$: 5.8° (*c* 0.1, MeOH); UV (MeOH) λ_max_ nm (log ε): 237 (3.47), 327 (3.22); IR (ATR) ν_max_ 1705, 1596, 1517, 1268, 1159, 1074 cm^−1^; NMR data: Table 2.

### 3.4. Acidic and Enzymatic Hydrolysis of **1**

Compound **1** (10.0 mg) was incubated together with *β*-glucosidase (25.0 mg), toluene (2 drops), and H_2_O (15.0 mL) in a CO_2_ incubator at 35 °C for 3 days. EtOH was added to the reaction mixture to stop the reaction and *β*-glucosidase was removed by filtration. Compound **2** (2.0 mg) was isolated from the hydrolysate by flash CC with Redi Sep-C18 cartridge (13 g, MeOH-H_2_O, from 50:50 to 80:20 *v/v*). Meanwhile, compound **1** (93.8 mg) was hydrolyzed with 2N HCl at 80 °C for one hour. The reaction was stopped by the addition of sodium bicarbonate and **1a** (8.0 mg) was isolated from the hydrolysate by flash CC with a Redi Sep-C18 cartridge (13 g, MeOH-H_2_O, 60:40 to 80:20, *v/v*). An enzymatic hydrolysis of **1a** (8.0 mg) was performed using the same method as **1** to give **1b** (1.5 mg).

### 3.5. Absolute Configurations of β-Glucose in **1**

The absolute configuration of *β*-glucose in **1** was determined by the previously reported method [21]. Pyridine (500 μL) and l-cysteine methyl ester hydrochloride (1.2 mg) were added in the hydrolysate and the mixture was heated at 60 °C for 1 h. *σ*-Tolyl isothiocyanate (100 μL) was added in the mixture and heated again at 60 °C for 1 hour. The reaction was analyzed directly by HPLC with a gradient system (10–50% of B, A: 0.1% (*v/v*) formic acid in water, B: 0.1% (*v/v*) formic acid in acetonitrile). The reaction mixture of **1** was detected at 27.4 min. At the same HPLC conditions, authentic l- and d-glucoses were detected at 26.8 and 27.4 min, respectively. Therefore, the absolute configuration of *β*-glucose in **1** was established as the d configuration.

### 3.6. Computational Methods

The 3D models of compounds **1b** and **5** were built by using Chem3D modeling. Conformational structure analysis, optimizations, and ECD calculations were performed as described previously [22,23].

### 3.7. Measurement of Cell Viability and NO Production

Cell viability and nitrite levels were measured using MTT and Griess reaction assays, respectively [24].

### 3.8. Measurement of PGE_2_

PGE_2_ levels in cell culture mediums were determined using EIA kits (R&D Systems, MN) as reported in the previous paper [24].

## 4. Conclusions

Four new eudesmene-type sesquiterpenoids (**1**, **3**, **6**, and **7**) were obtained from a 95% EtOH extract of the leaves of *Aster koraiensis* by repeated chromatography, along with five known compounds (**2**, **4**, **5, 8**, and **9**). The chemical structures of the four new compounds and absolute configurations of the known compounds **2**, **4**, and **5** were established by their spectroscopic data (HR-MS, 1D- & 2D-NMR, and ECD) measurement and by acidic and enzymatic hydrolysis. Among the isolates, the new compound **7** exhibited weak inhibitory activities on both NO and PGE_2_ production. The compounds found in this study do not appear to contribute to the anti-inflammatory activity of the extract from which they were isolated. Thus, compounds with higher activity in the leaves of *A. koraiensis* needs to be identified through further studies.

## Figures and Tables

**Table 1:** ^1^H and ^13^C nuclear magnetic resonance (NMR) spectroscopic data of **1** and **3** (*δ* in ppm, C_5_D_5_N, 500, and 125 MHz).

Position ^a^	1		3	
	*δ*_H_ Multi (*J* in Hz)	*δ*_C_	*δ*_H_ Multi (*J* in Hz)	*δ*_C_
1	4.18 d (9.5)	78.4	4.17 m	79.4
2	2.06 m/2.25 td (13.0, 11.0)	30.3	2.73 q (12.0)/2.21 m	28.5
3	5.30 br s	120.4	4.98 br s	120.7
4	-	136.7	-	136.7
5	1.83 br s	52.3	1.83 s	52.3
6	4.64 m	76.6	4.66 br s	80.2
7	0.99 m	51.7	1.37 s	51.6
8	2.37 m/2.53 m	29.4	2.48 m	27.3
9	4.08 m	80.2	4.16 m	85.0
10	-	43.0	-	42.9
11	2.37 m	29.0	-	72.6
12	0.85 d (6.5)	21.9	1.58 s	29.2
13	1.11 d (6.0)	21.9	1.38 s	29.5
14	1.64 s	10.7	1.68 s	10.9
15	2.10 s	22.0	1.93 s	22.4
Glc-1’	4.92 d (8.0)	104.6	4.87 d (8.0)	105.5
Glc-2’	3.96 t (8.5)	76.2	3.97 dd (8.0, 8.0)	75.7
Glc-3’	4.10 m	78.4	4.21–4.15	78.8
Glc-4’	4.15 m	72.1	4.01 dd (9.5)	72.1
Glc-5’	3.86 m	77.4	3.82	77.6
Glc-6’	4.31 t (6.0)/4.47 dd (11.5, 3.0)	63.4	4.38 m/4.21–4.15	64.0
Glc-1”	4.99 d (8.0)	102.1	4.99 d (8.0)	102.2
Glc-2”	4.64 m	75.4	3.90 dd (8.0, 8.0)	75.5
Glc-3”	4.27 t (9.0)	78.1	4.21–4.15	78.8
Glc-4”	4.14 t (4.0)	71.6	4.10 m	71.5
Glc-5”	4.18 d (9.5)	86.4	4.21–4.15	79.3
Glc-6”	4.41 dd (12.0, 6.0)/4.64 m	62.9	4.68 m/4.42 m	63.4

**^a^** All assignments were supported with ^1^H-^1^H correlation spectroscopy (COSY), ^1^H-^13^C heteronuclear single quantum coherence spectroscopy (HSQC), and ^1^H-^13^C heteronuclear multiple bond correlation (HMBC) experiments.

**Table 2:** ^1^H and ^13^C NMR spectroscopic data of **6** and **7** (*δ* in ppm, CD_3_OD, 500, and 125 MHz).

Position ^a^	6		7	
	*δ*_H_ Multi (*J* in Hz)	*δ*_C_	*δ*_H_ Multi (*J* in Hz)	*δ*_C_
1	4.87 overlapped	80.8	4.89 overlapped	80.5
2	2.22 m/2.09 m	29.6	2.25 m/2.09 m	29.7
3	5.29 br s	120.4	5.30 br s	120.7
4		136.2		136.0
5	2.10 br s	52.3	2.11 br s	52.4
6	4.46 s	76.0	4.47 s	76.0
7	1.18 m	51.5	1.18 m	51.5
8	1.98 m	29.1	1.93 m	29.1
9	4.92 overlapped	80.3	4.98 overlapped	80.4
10		42.2		42.3
11	1.89 m	29.0	1.98 m	29.1
12	1.02 d (6.5)	21.9	1.02 d (6.5)	21.9
13	0.93 d (6.5)	21.8	0.92 d (6.5)	21.8
14	1.26 s	11.8	1.37 s	11.9
15	1.88 s	22.0	1.87 s	22.0
Glc-1’	4.38 d (8.0)	104.5	4.39 d (8.0)	104.5
Glc-2’	3.14 br t (8.5)	76.2	3.16 br t (8.0)	76.2
Glc-3’	3.32 m	78.5	3.32 m	78.5
Glc-4’	3.31 m	72.1	3.31 m	72.1
Glc-5’	3.21 m	77.5	3.21 m	77.6
Glc-6’	3.83 dd (11.5, 2.5)/3.68 dd(11.5, 5.5)	63.3	3.83 dd (11.5, 2.5)/3.69 dd(11.5, 5.5)	63.3
1”		127.9		124.5
2”	7.70 d (8.5)	134.2	7.22 d (2.0)	111.9
3”	6.76 d (8.5)	116.0	7.08 dd (8.5, 2.0)	120.7
4”		160.3		149.6
5”	6.76 d (8.5)	116.0	6.81 d(8.5)	116.7
6”	7.70 d (8.5)	134.2		150.8
7”	6.89 d (12.5)	145.7	7.61 d (16.0)	147.0
8”	5.80 d (12.5)	117.7	6.38 d (16.0)	116.7
9”		167.8		168.8
OCH_3_	-	-	3.91 s	56.7

**^a^** All assignments were supported with COSY, HSQC, and HMBC experiments.

**Table 3:** The cytotoxicities and inhibitory activities of **1**–**9** obtained from the leaves of *A. koraiensis* on LPS-induced NO and PGE_2_ production in RAW 264.7 macrophages.

Compound	Cell Viabilities (%) ^a^	Inhibition Rate (%) ^a^ [IC_50_ (μM)]	
		NO	PGE_2_
**1**	102.46	1.79 [>100]	0 [>100]
**2**	105.31	3.20 [>100]	0 [>100]
**3**	94.03	4.66 [>100]	10.48 [>100]
**4**	102.18	1.31 [>100]	6.15 [>100]
**5**	103.83	8.39 [>100]	0 [>100]
**6**	107.78	7.01 [>100]	2.16 [>100]
**7**	89.35	53.12 [95.7]	41.26 [111.6]
**8**	93.38	6.25 [>100]	1.27 [>100]
**9**	100.41	4.64 [>100]	20.42 [>100]

**^a^** Cells were pretreated with **1**–**9** (100 μM) and LPS (1 μg/mL) for 1 h, and incubated for 24 h.

## Supplementary Materials

The following are available online at https://www.mdpi.com/2223-7747/9/12/1811/s1, General Experimental Procedure, The HR-ESI-MS, ^1^H-NMR (500 MHz, C_5_D_5_N), ^13^C-NMR (125 MHz, C_5_D_5_N), HSQC, COSY, HMBC, and NOESY spectra of **1** (Figures S1~S8), The HR-ESI-MS, ^1^H-NMR (500 MHz, C_5_D_5_N), ^13^C-NMR (125 MHz, C_5_D_5_N), HSQC, COSY, HMBC, and NOESY spectra of **3** (Figures S9~S15), The HR-ESI-MS, ^1^H-NMR (500 MHz, CD_3_OD), ^13^C-NMR (125 MHz, CD_3_OD), HSQC, COSY, HMBC, and NOESY spectra of **6** (Figures S16~S21), The HR-ESI-MS, ^1^H-NMR (500 MHz, CD_3_OD), ^13^C-NMR (125 MHz, CD_3_OD), COSY, HMBC, and NOESY spectra of **7** (Figures S22~S27).
