# Supplementary material for: New Eudesmane-Type Sesquiterpene Glycosides from the Leaves of Aster koraiensis
Source: Plants (Basel). 2020 Dec 21;9(12):1811. doi: 10.3390/plants9121811 (PMC7766762; doi:10.3390/plants9121811)

Supplementary metarials

Contents

General Experimental procedure
Figure S1. The HR-ESI-MS spectrum of compound **1**
Figure S2. The ^{1}H-NMR (500 MHz, C_5D_5N) spectrum of compound **1**
Figure S3. The ^{13}C-NMR (125 MHz, C_5D_5N) spectrum of compound **1**
Figure S4. The HSQC spectrum of compound **1**
Figure S5. The COSY spectrum of compound **1**
Figure S6. The HMBC spectrum of compound **1**
Figure S7. The NOESY spectrum of compound **1**
Figure S8. The HR-ESI-MS spectrum of compound **3**
Figure S9. The ^{1}H-NMR (500 MHz, C_5D_5N) spectrum of compound **3**
Figure S10. The ^{13}C-NMR (125 MHz, C_5D_5N) spectrum of compound **3**
Figure S11. The HSQC spectrum of compound **3**
Figure S12. The COSY spectrum of compound **3**
Figure S13. The HMBC spectrum of compound **3**
Figure S14. The NOESY spectrum of compound **3**
Figure S15. The HR-ESI-MS spectrum of compound **6**
Figure S16. The ^{1}H-NMR (500 MHz, CD_3OD) spectrum of compound **6**
Figure S17. The ^{13}C-NMR (125 MHz, CD_3OD) spectrum of compound **6**
Figure S18. The HSQC spectrum of compound **6**
Figure S19. The COSY spectrum of compound **6**
Figure S20. The HMBC spectrum of compound **6**
Figure S21. The NOESY spectrum of compound **6**
Figure S22. The HR-ESI-MS spectrum of compound **7**
Figure S23. The ^{1}H-NMR (500 MHz, CD_3OD) spectrum of compound **7**
Figure S24. The ^{13}C-NMR (125 MHz, CD_3OD) spectrum of compound **7**
Figure S25. The COSY spectrum of compound **7**
Figure S26. The HMBC spectrum of compound **7**
Figure S27. The NOESY spectrum of compound **7**

General Experimental procedure

UV spectra were evaluated on Optizen pop (Mecasys, Daejeon, Korea). MPA 100 (Stanford research systems, Sunnyvale, CA, USA) was used to measure melting points in open capillary tubes. Optical rotations were obtained on a Jasco P-2000 polarimeter (JASCO, Tokyo, Janpan), using a 10-cm microcell. JEOL (JEOL, Tokyo, Japan) 500 MHz was used for obtaining NMR spectra. HR-Mass spectra were obtained by a Q-TOF micro mass spectrometer (Waters, Milford, Massachusetts, USA). TLC analyses were performed on Silica gel 60 F_{254} (Merck, Kenilworth, MA, USA) and RP-18 F_{254S} (Merck) plates. Compounds were visualized by dipping plates into 20% (v/v) H_2SO_4 reagent (Samchun) and then heated at 110°C for 5-10 min. Agilent Cary 630 FTIR (Agilent Technologies, Santa Clara, CA, USA) was applied to obtain IR spectrum. Sephadex LH-20 (Amersham Pharmacia Biotech, Buckinghamshire, United Kingdom), Silica gel (Merck 60A, 230-400 mesh ASTM), Diaion HP-20 (Mitsubishi, Tokyo, Japan), and reversed-phase silica gel (YMC Co., ODS-A 12 nm S-150 μm) were used for column chromatography. Pre-packed cartridges, Redi Sep-Silica (12 g, 24 g, 40 g, Teledyne Isco) Combiflash PF, with Redi Sep-Silica (12 g, 24 g, 40 g, Teledyne Isco) and Redi Sep-C18 (13 g, 26 g, 43 g, 130 g, Teledyne Isco) were used for flash chromatography. HPLC was performed using Waters purification system (1525 pump, PDA 1996 detector) with Gemini NX-C18 110A column (250 × 21.2mm i.d. 5 μm, Phenomenex, Torrance, CA, USA). Flash chromatography was performed using the flash purification system (Combi Flash Rf, Teledyne Isco). Before chromatographic separations, all solvents used for this study were distilled.

Figure S1. The HR-ESI-MS spectrum of compound **1**

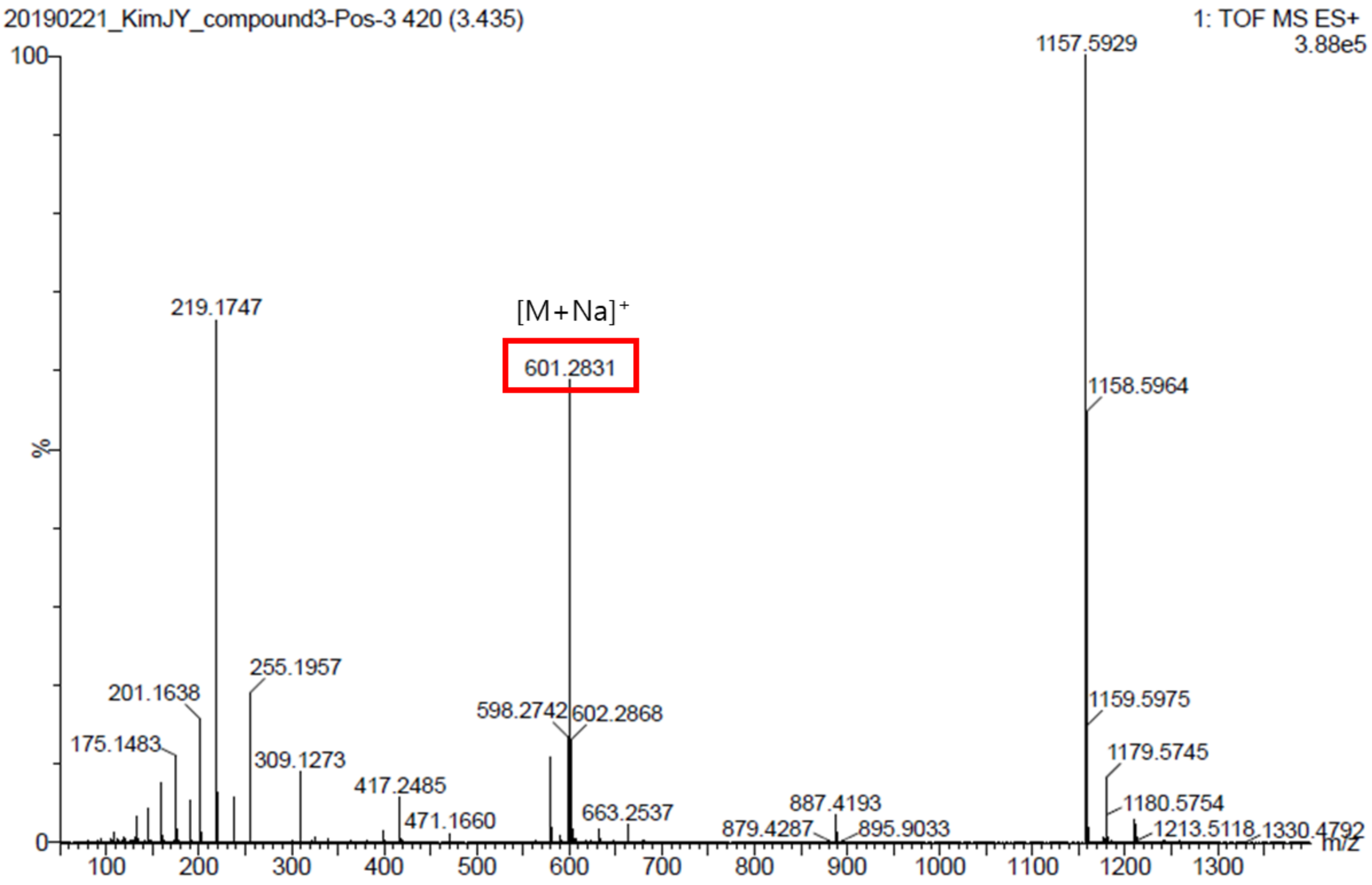

Figure S2. The ^{1}H-NMR (500 MHz, C_5D_5N) spectrum of compound **1**

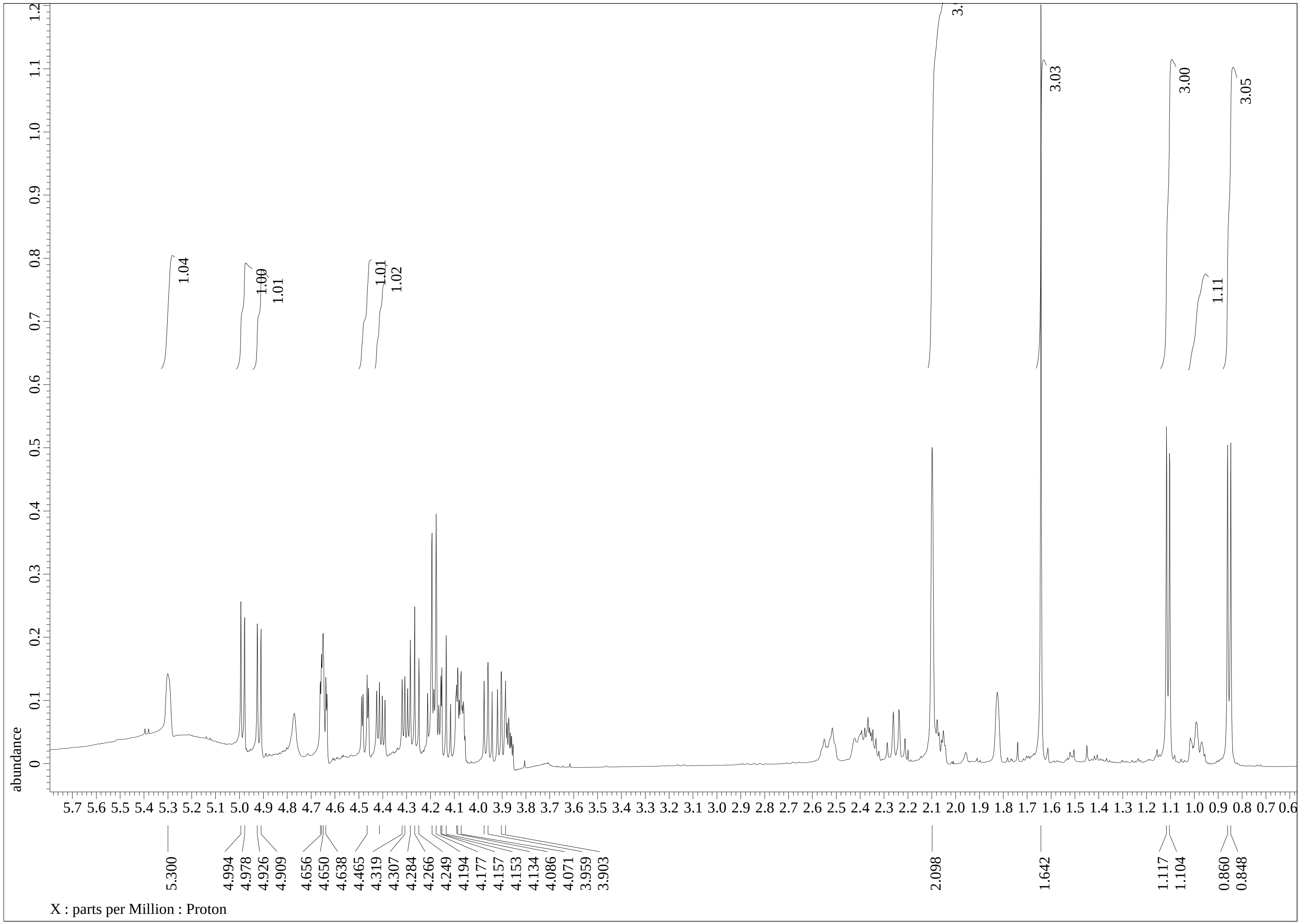

Figure S3. The ^{13}C-NMR (125 MHz, C_5D_5N) spectrum of compound **1**

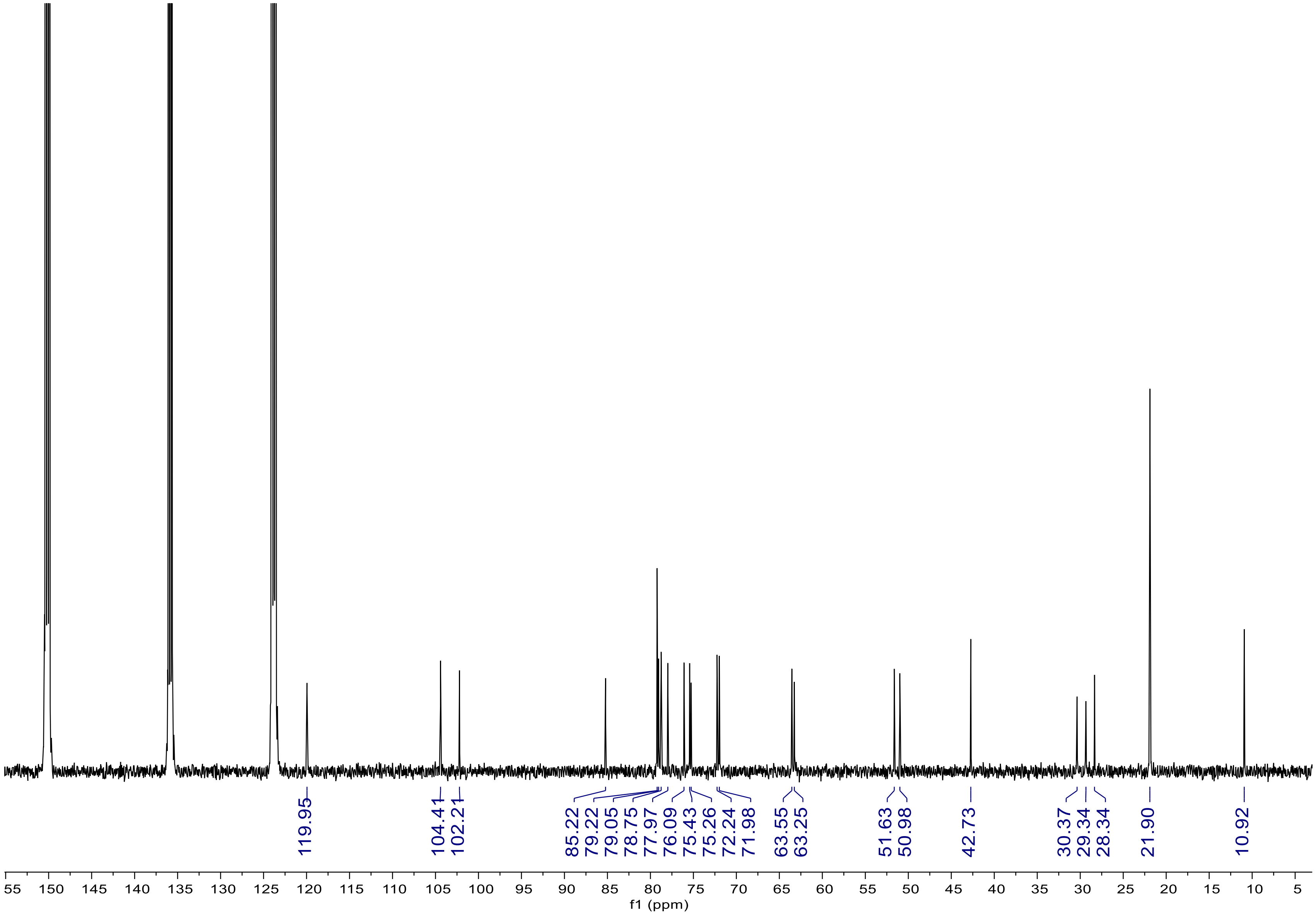

Figure S4. The HSQC spectrum of compound **1**

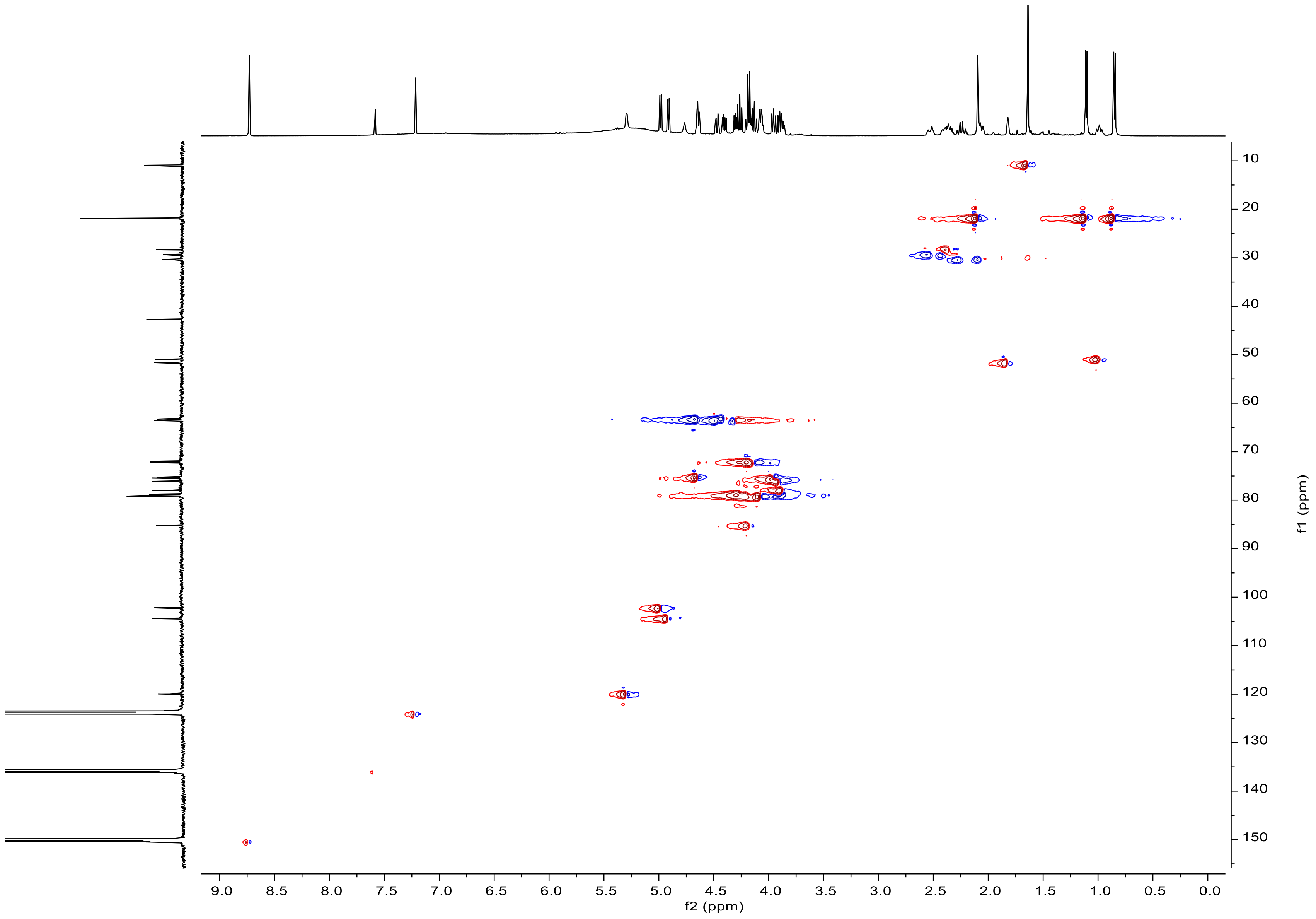

Figure S5. The COSY spectrum of compound **1**

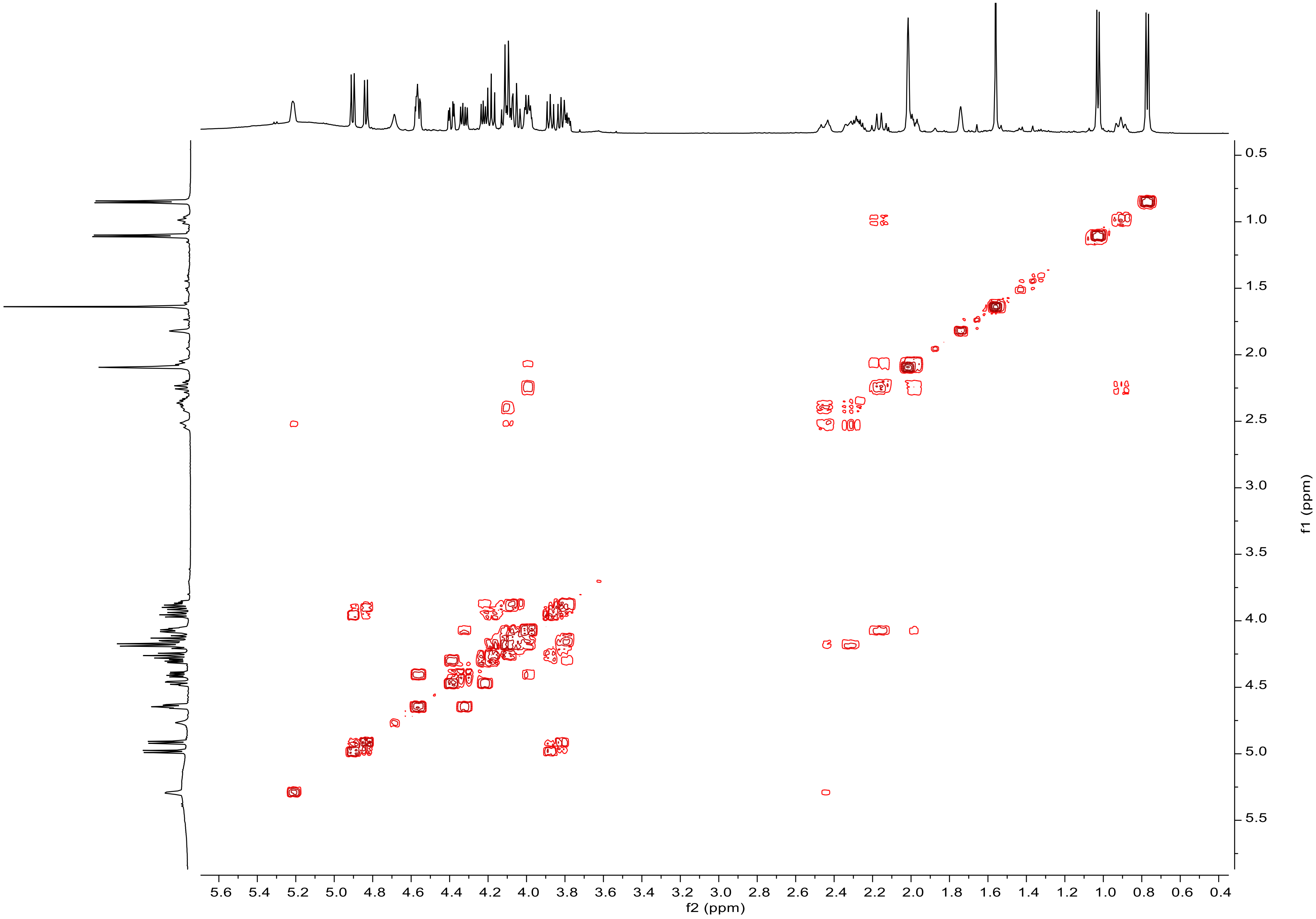

Figure S6. The HMBC spectrum of compound **1**

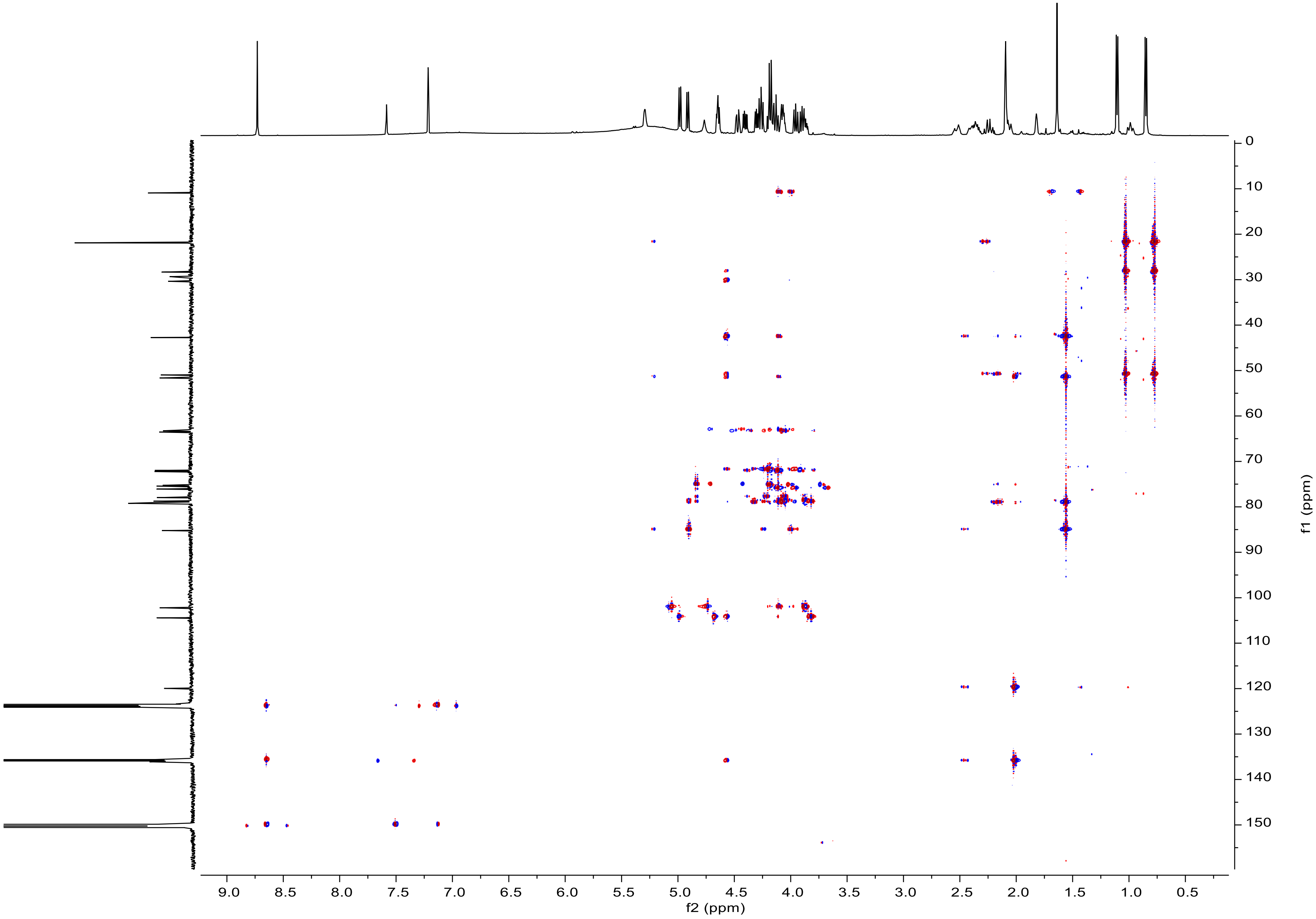

Figure S7. The NOESY spectrum of compound **1**

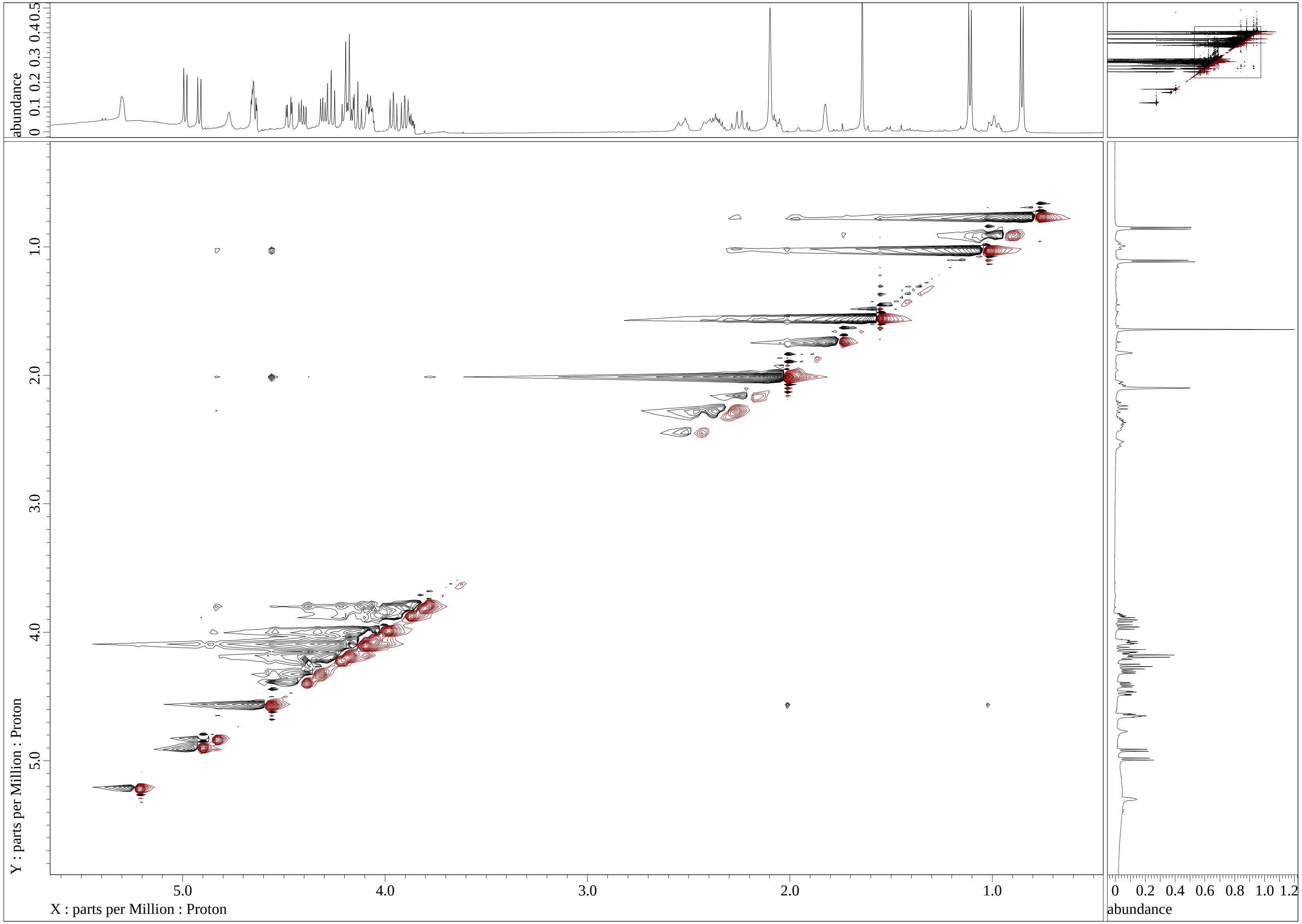

Figure S8. The HR-ESI-MS spectrum of compound **3**

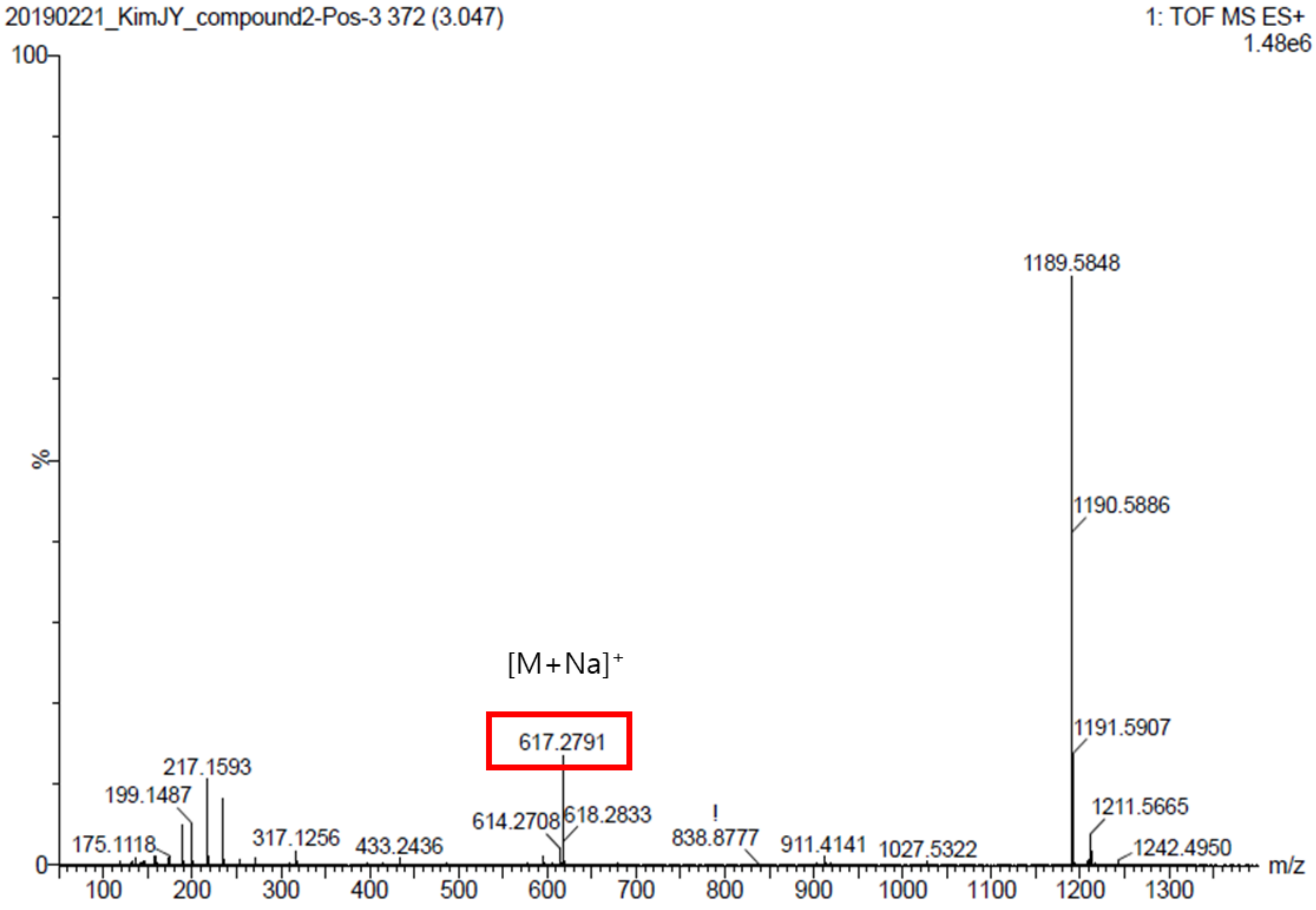

Figure S9. The ^{1}H-NMR (500 MHz, C_5D_5N) spectrum of compound **3**

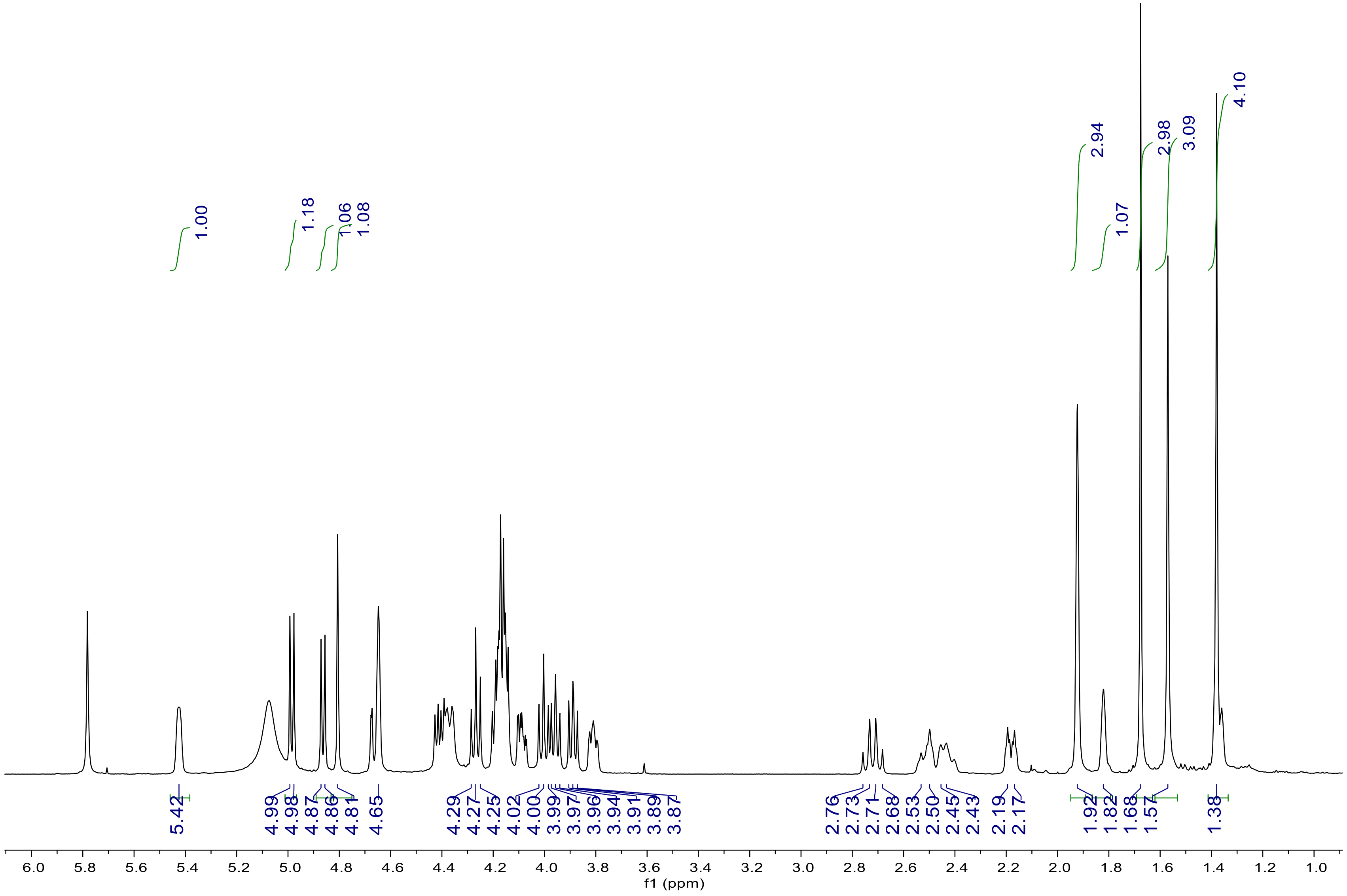

Figure S10. The ^{13}C-NMR (125 MHz, C_5D_5N) spectrum of compound **3**

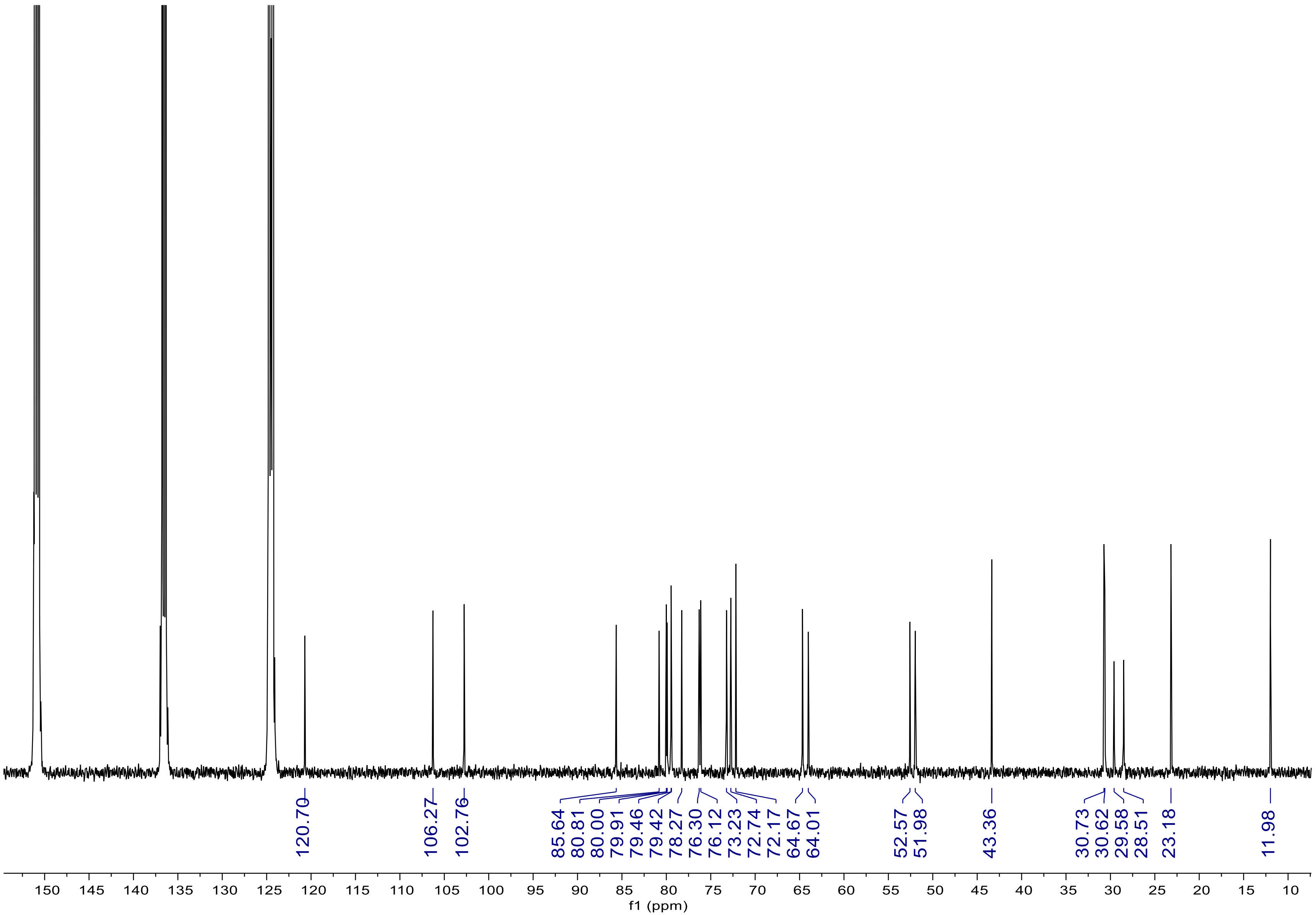

Figure S11. The HSQC spectrum of compound **3**

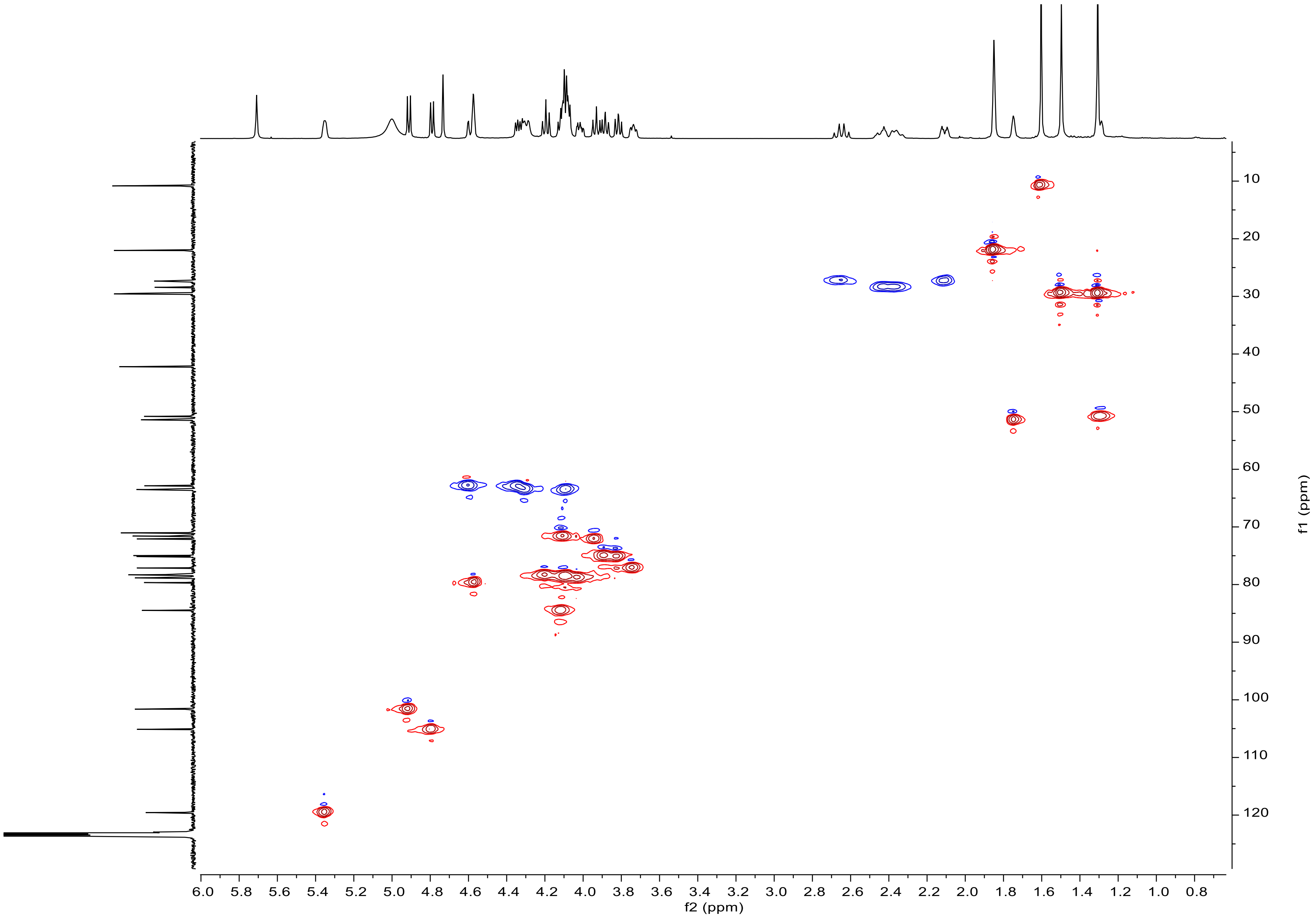

Figure S12. The COSY spectrum of compound **3**

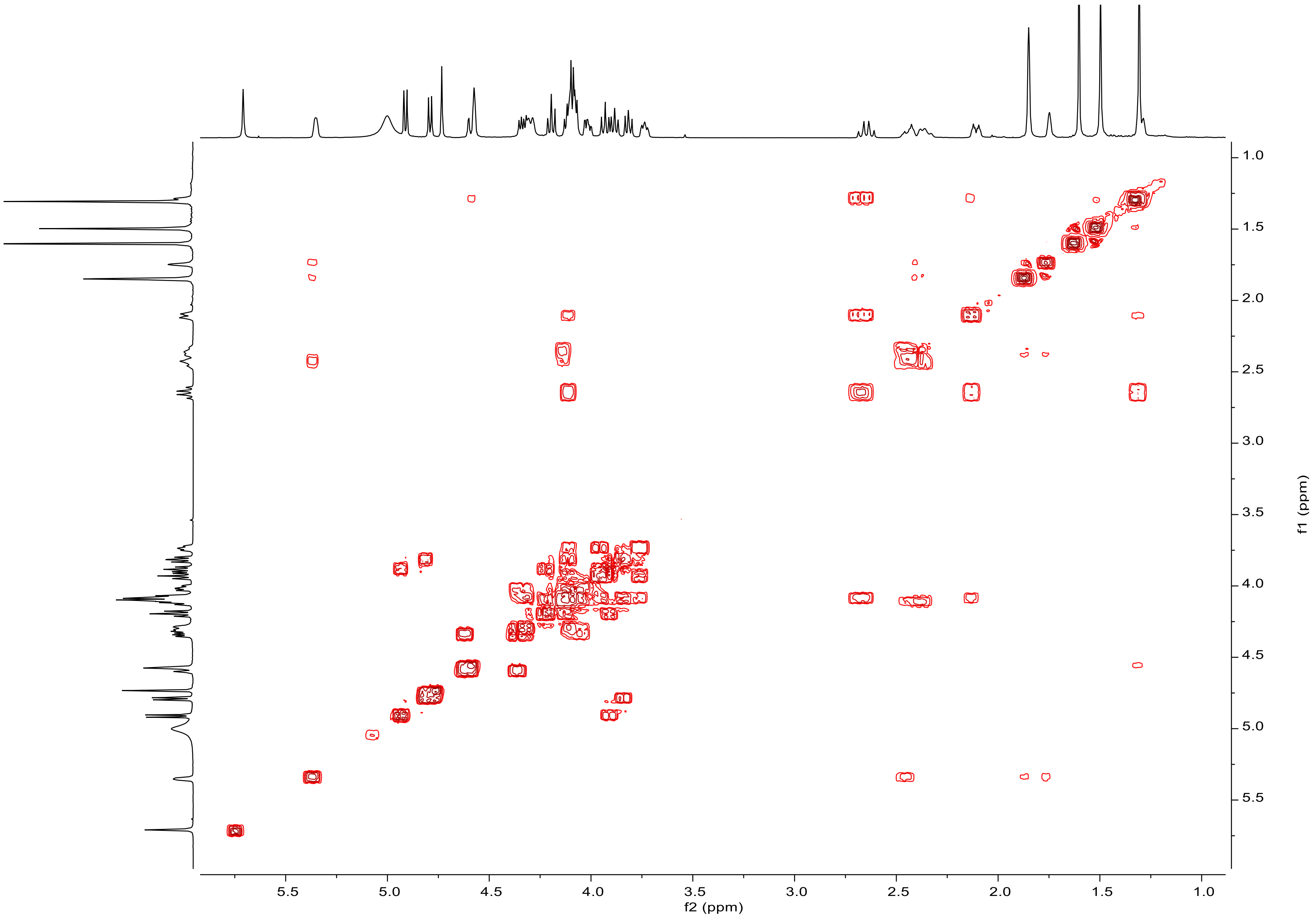

Figure S13. The HMBC spectrum of compound **3**

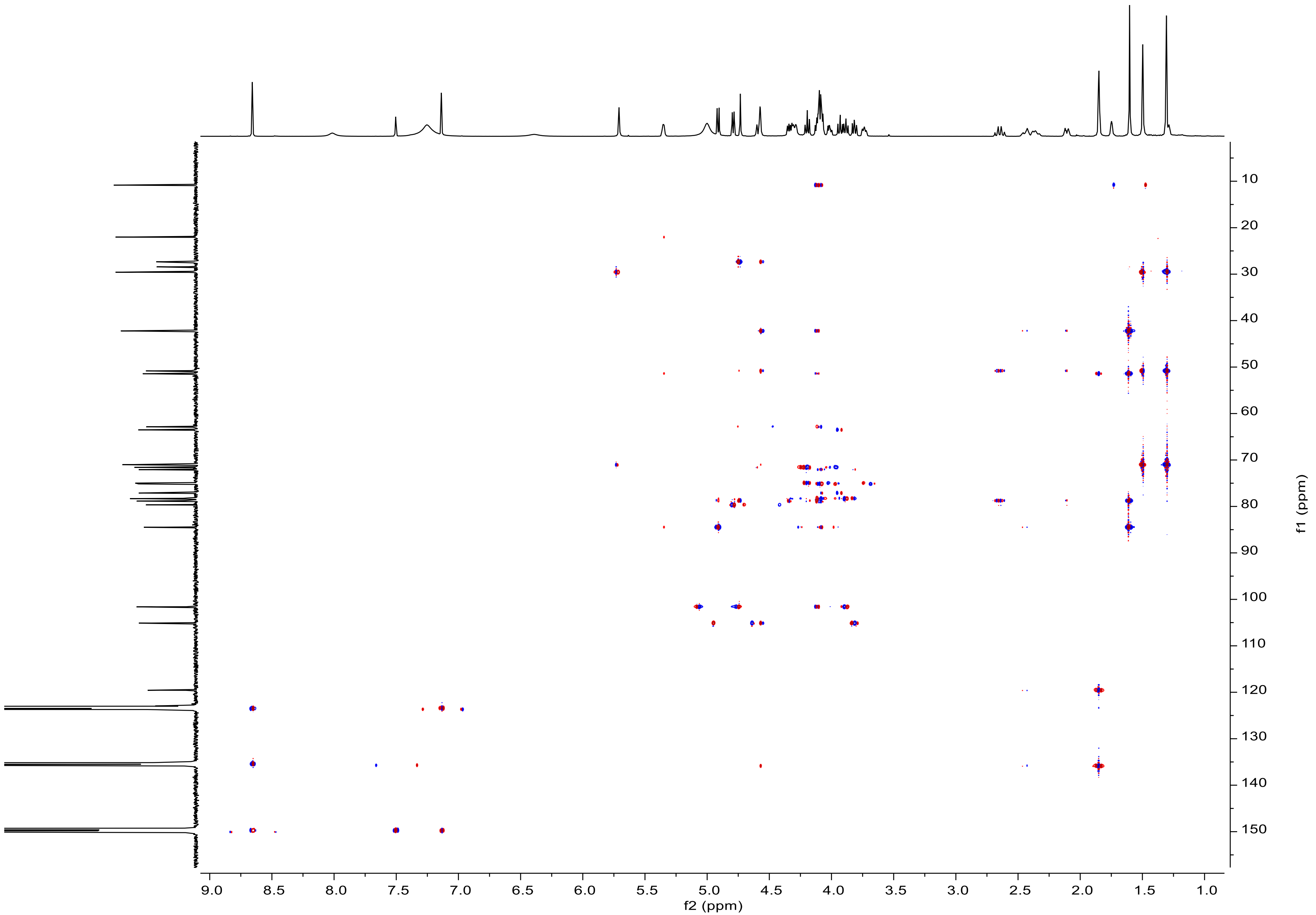

Figure S14. The NOESY spectrum of compound **3**

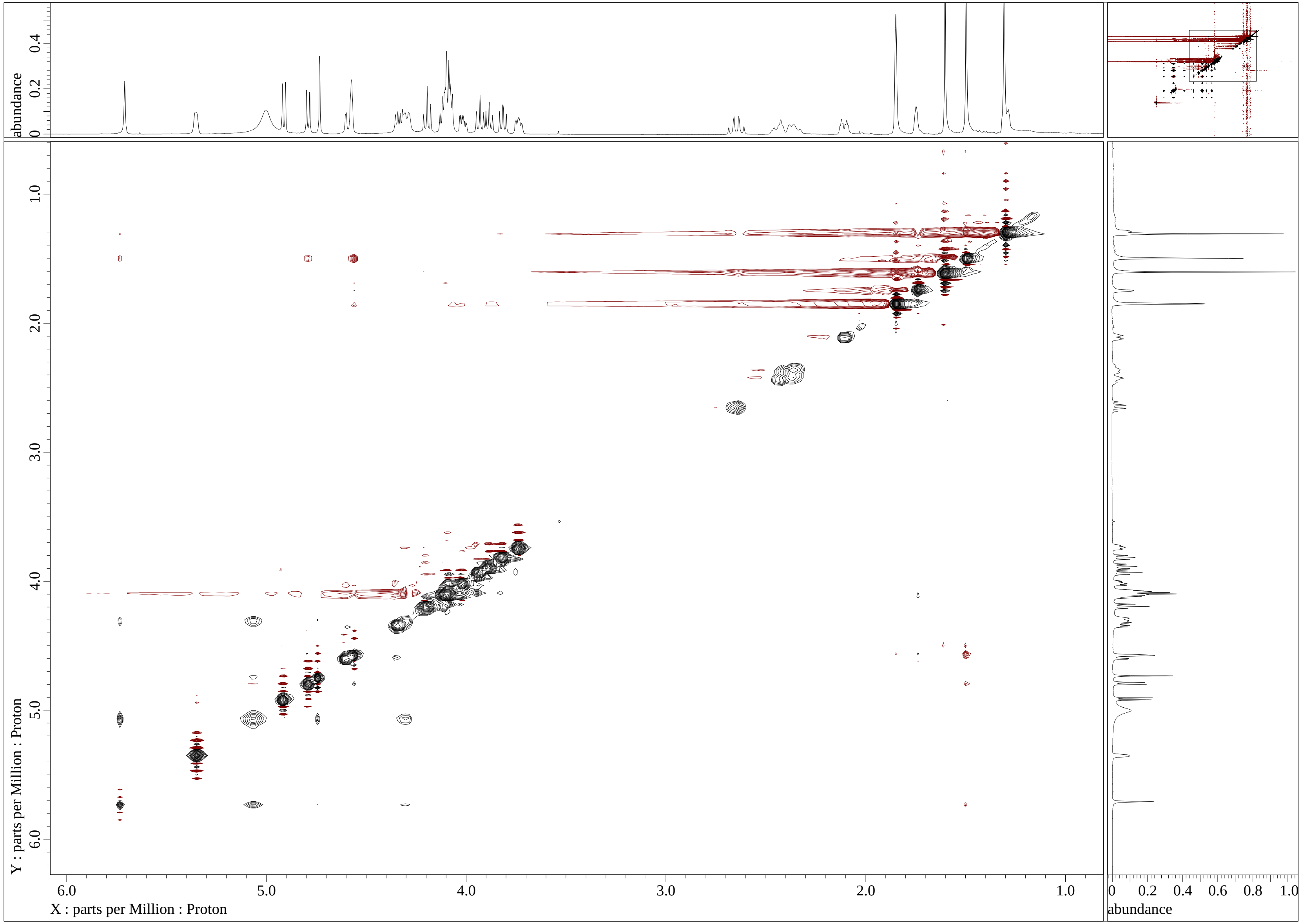

Figure S15. The HR-ESI-MS spectrum of compound **6**

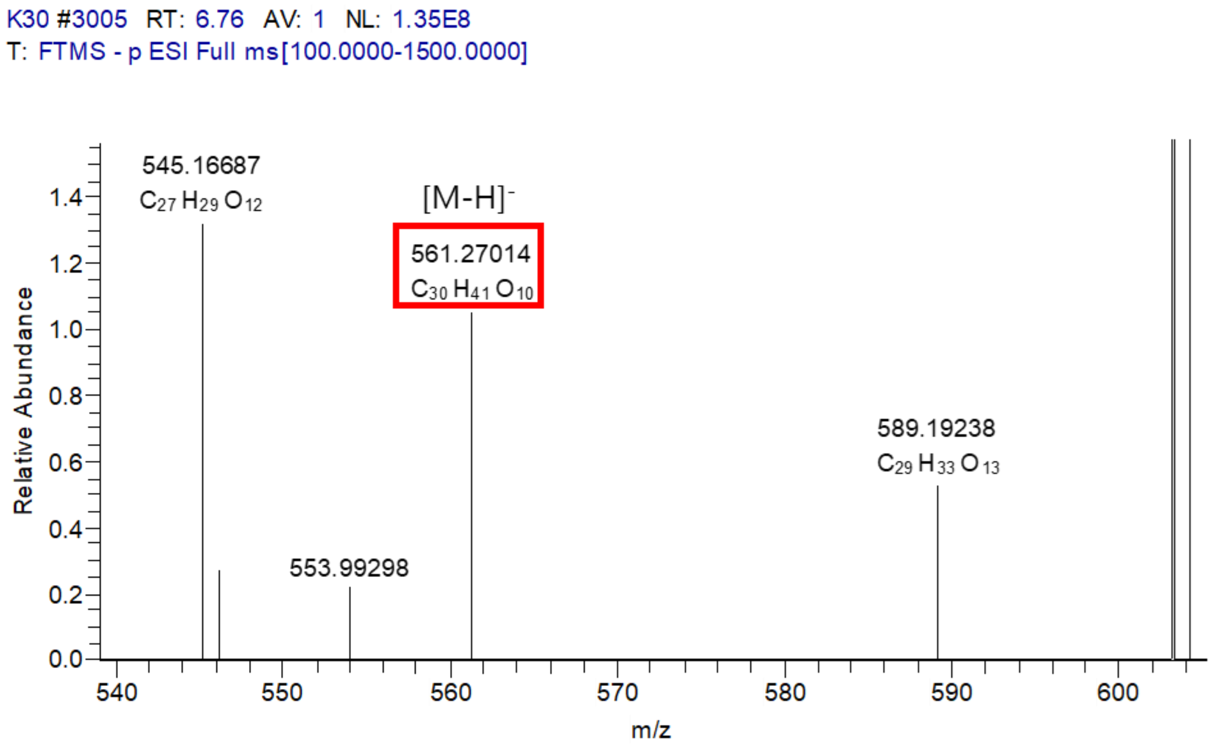

Figure S16. The ^{1}H-NMR (500 MHz, CD_3OD) spectrum of compound **6**

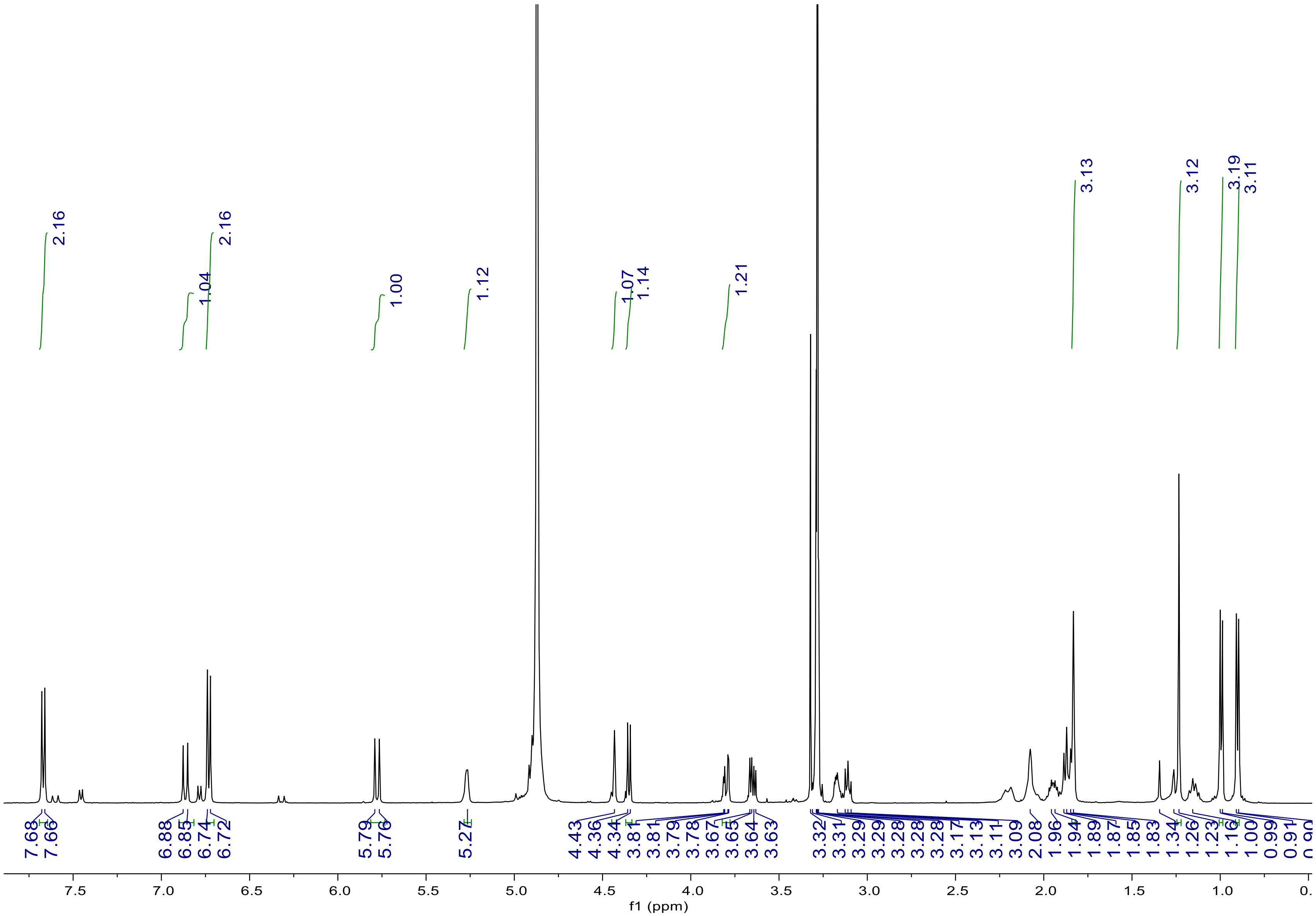

Figure S17. The ^{13}C-NMR (125 MHz, CD_3OD) spectrum of compound **6**

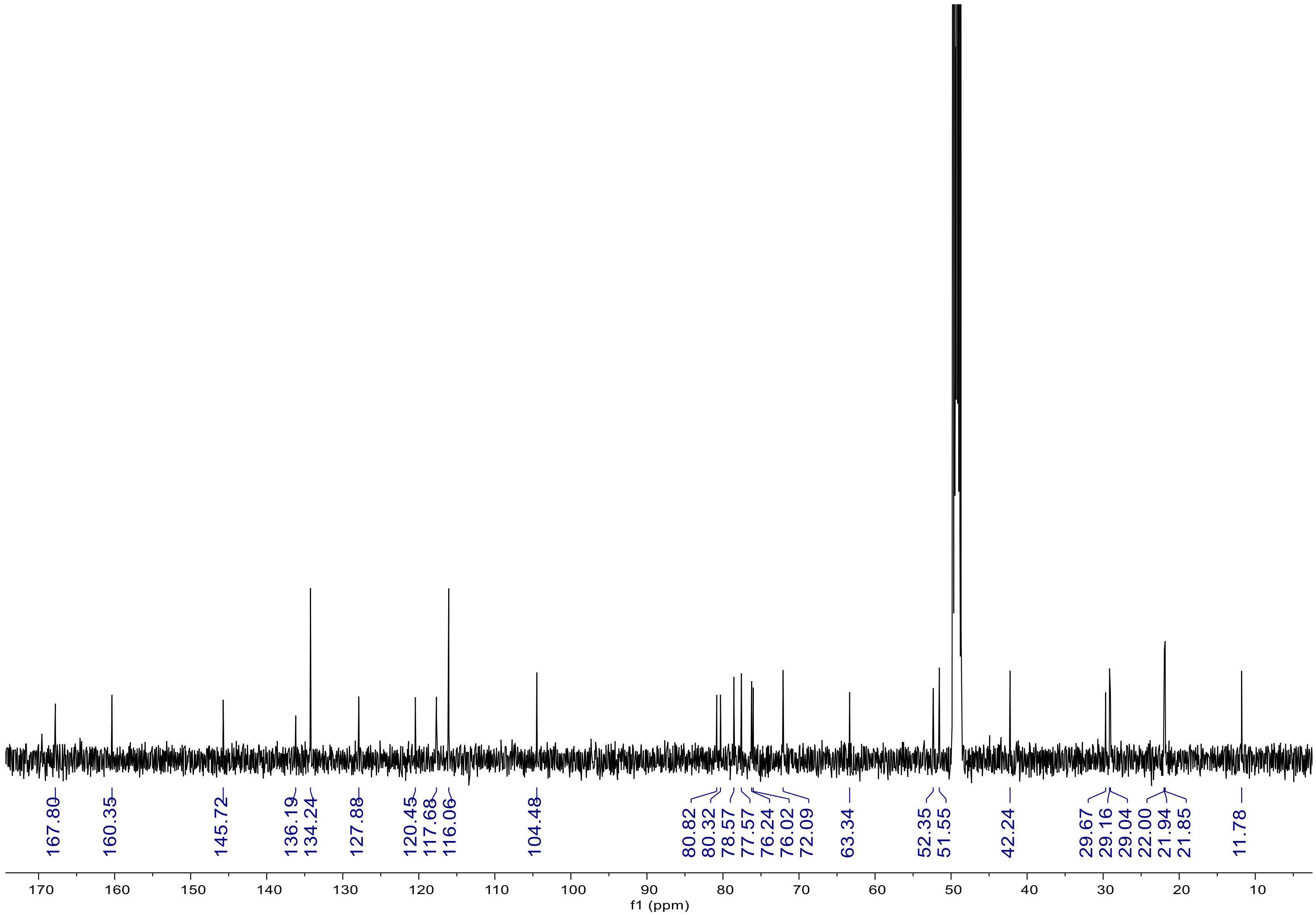

Figure S18. The HSQC spectrum of compound **6**

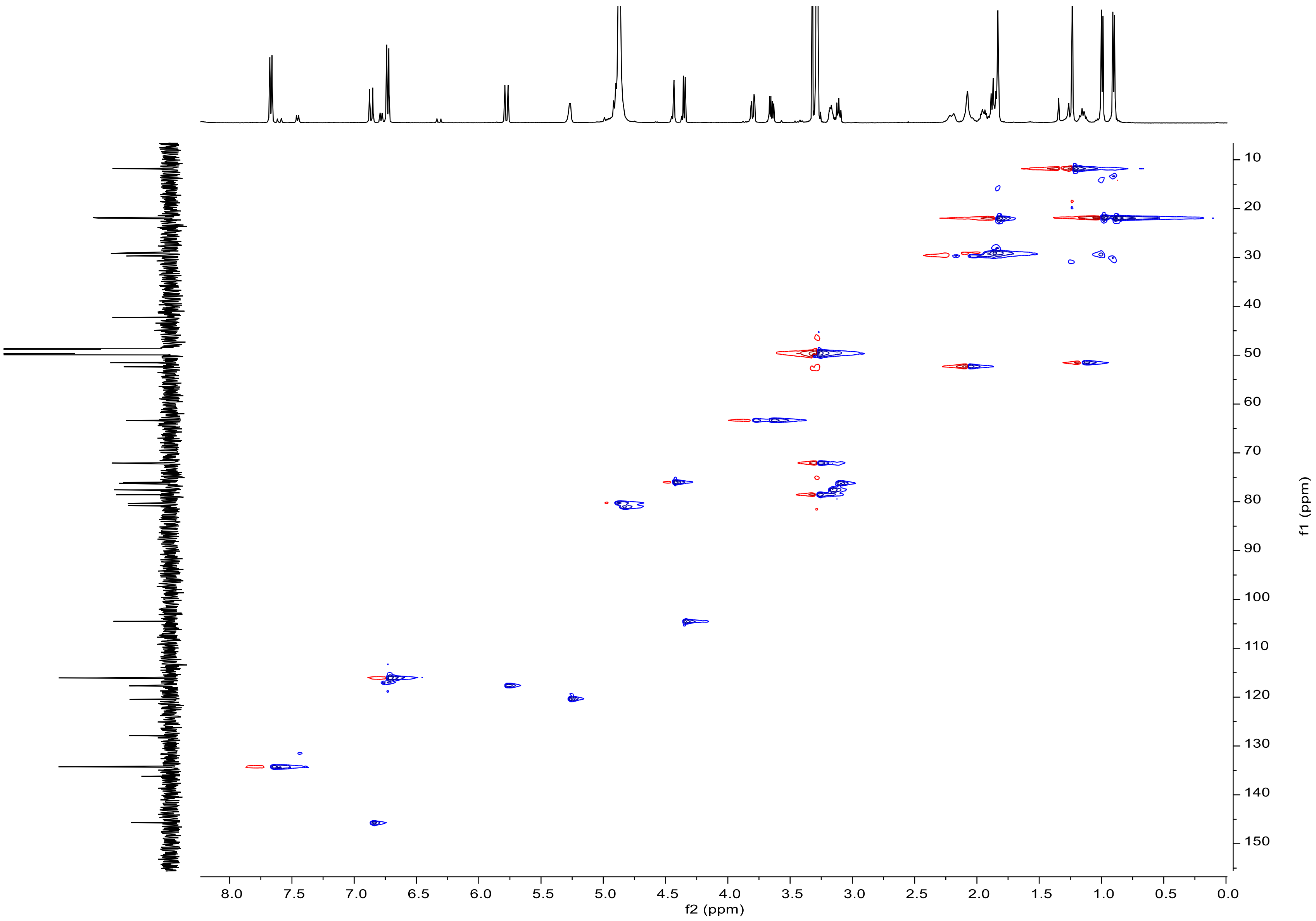

Figure S19. The COSY spectrum of compound **6**

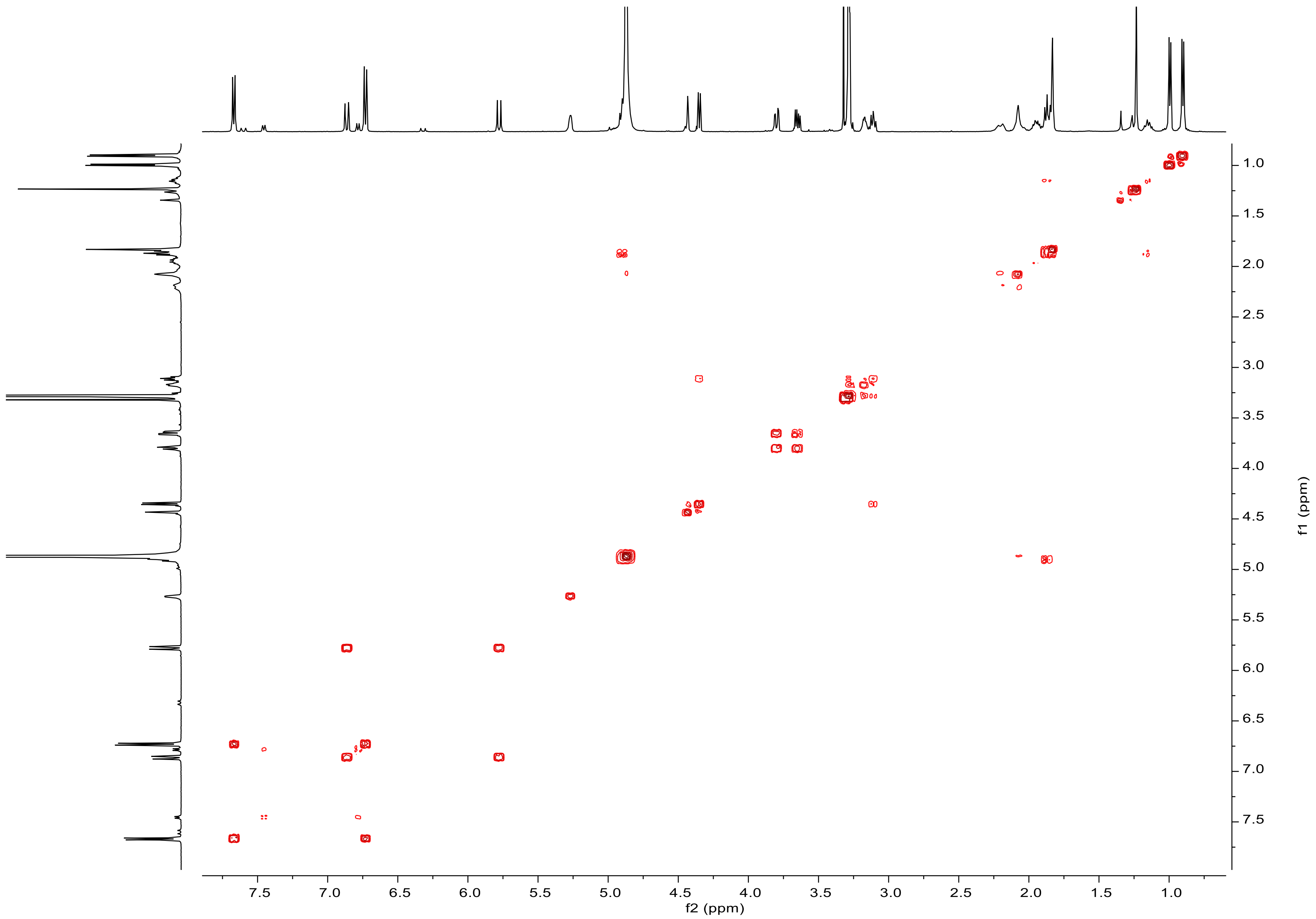

Figure S20. The HMBC spectrum of compound **6**

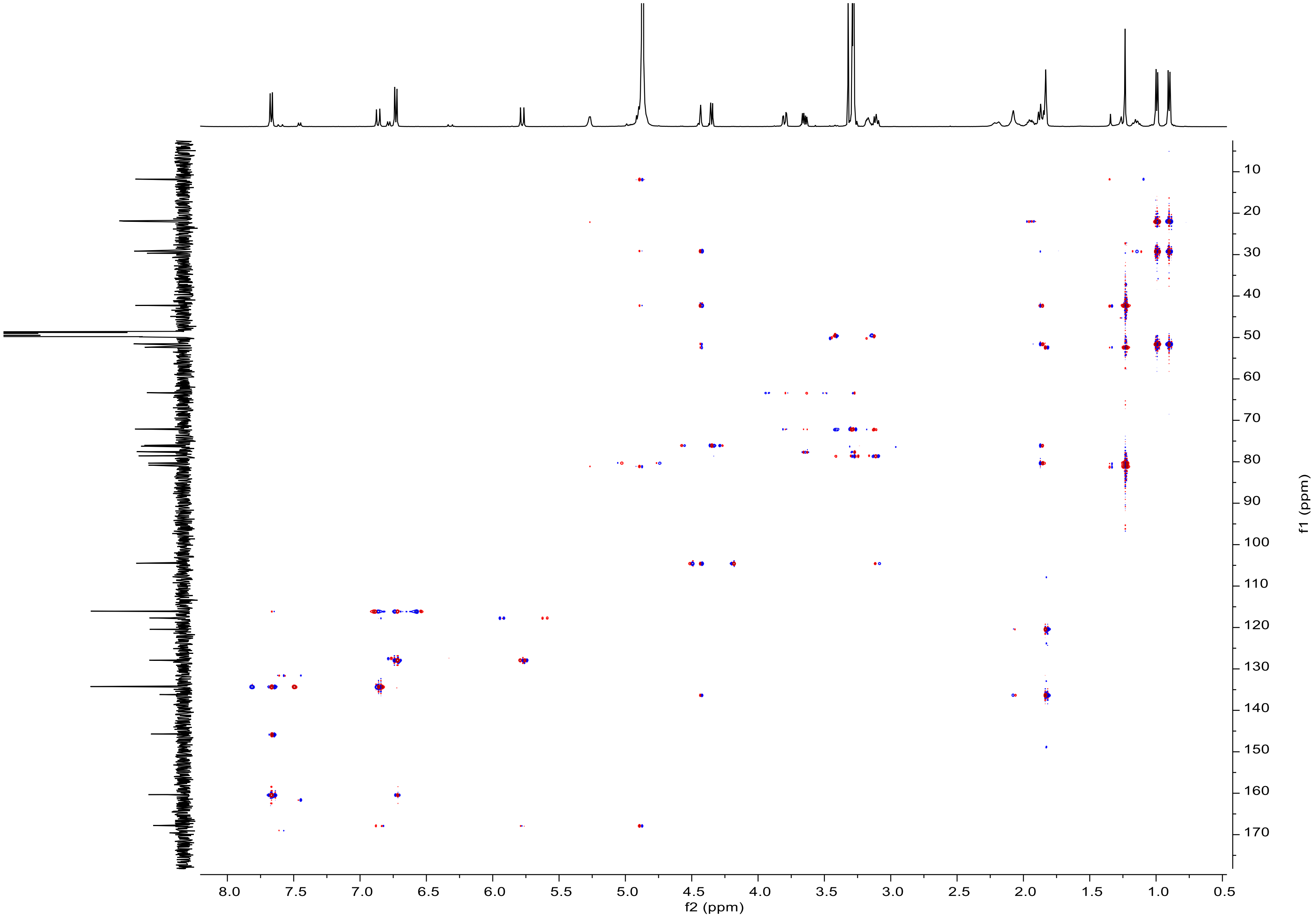

Figure S21. The NOESY spectrum of compound **6**

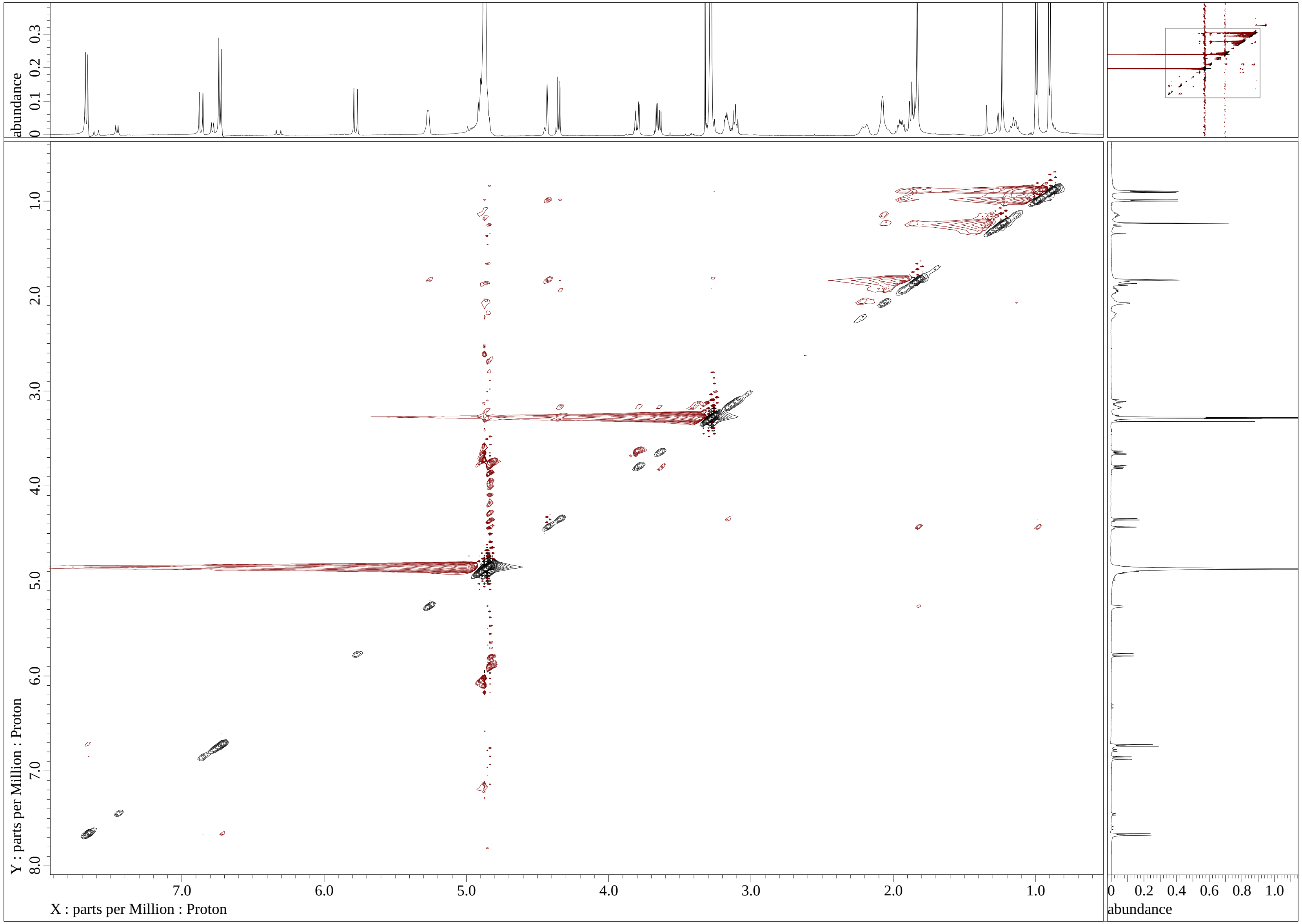

Figure S22. The HR-ESI-MS spectrum of compound **7**

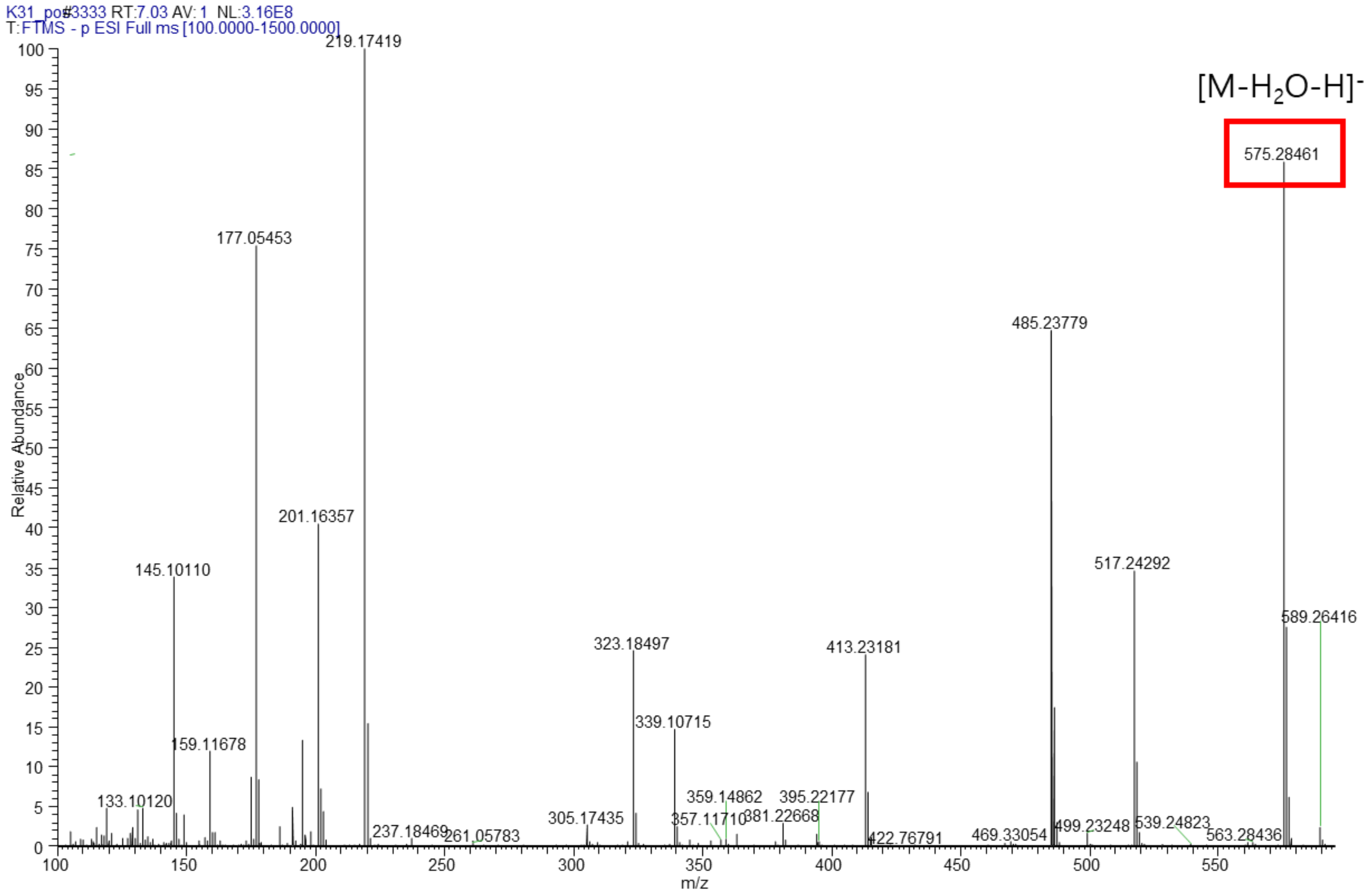

Figure S23. The ^{1}H-NMR (500 MHz, CD_3OD) spectrum of compound **7**

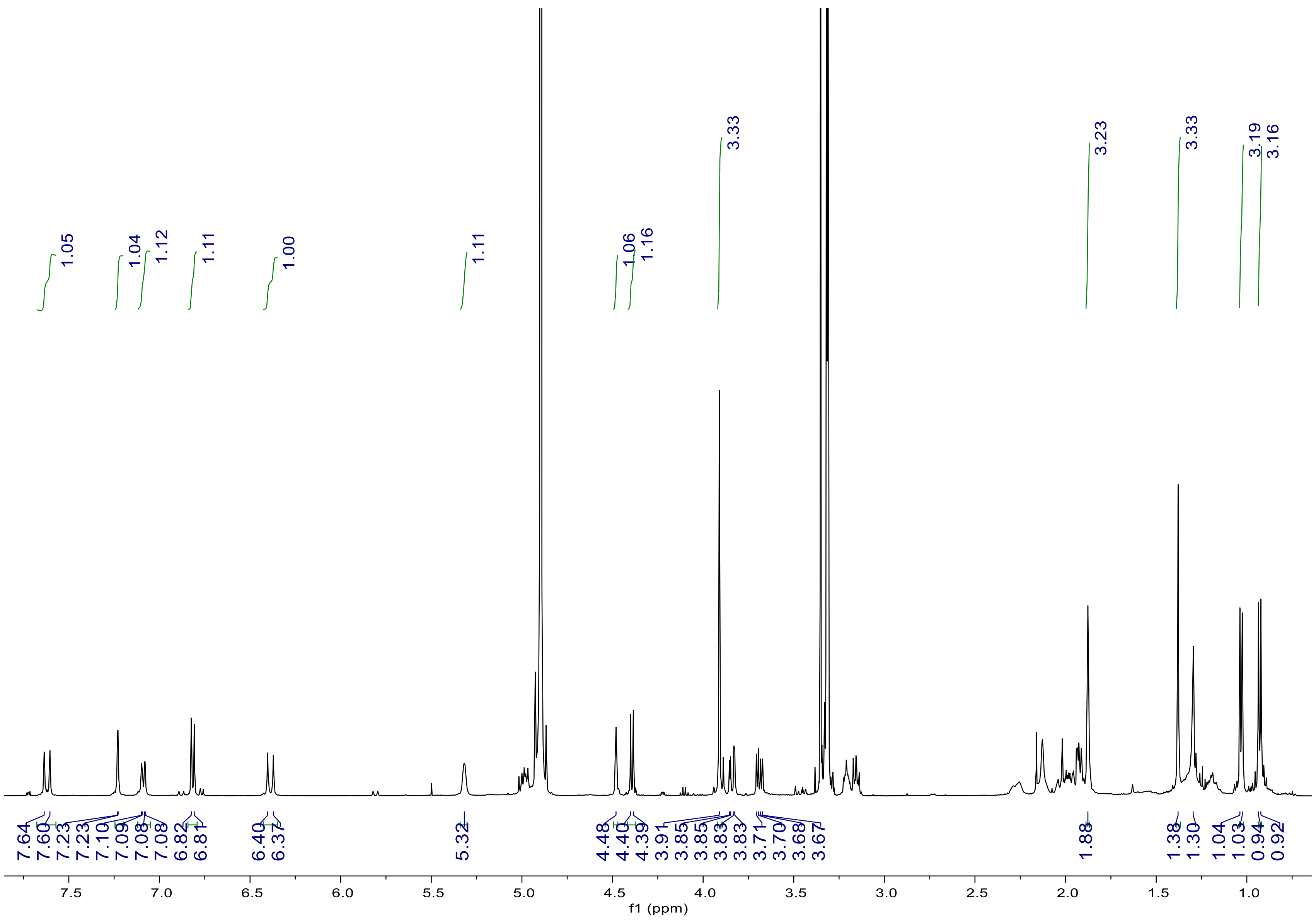

Figure S24. The ^{13}C-NMR (125 MHz, CD_3OD) spectrum of compound **7**

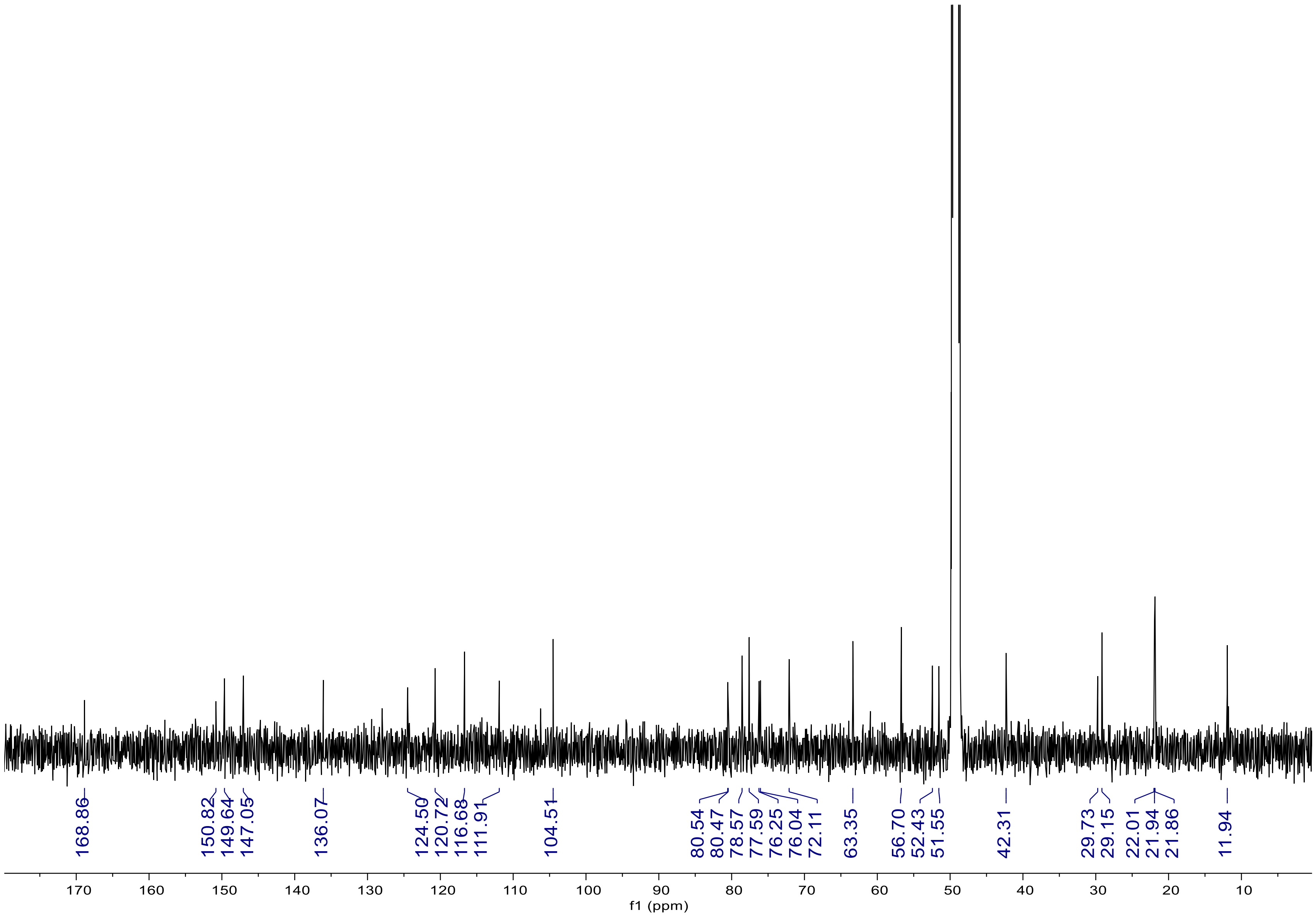

Figure S25. The COSY spectrum of compound **7**

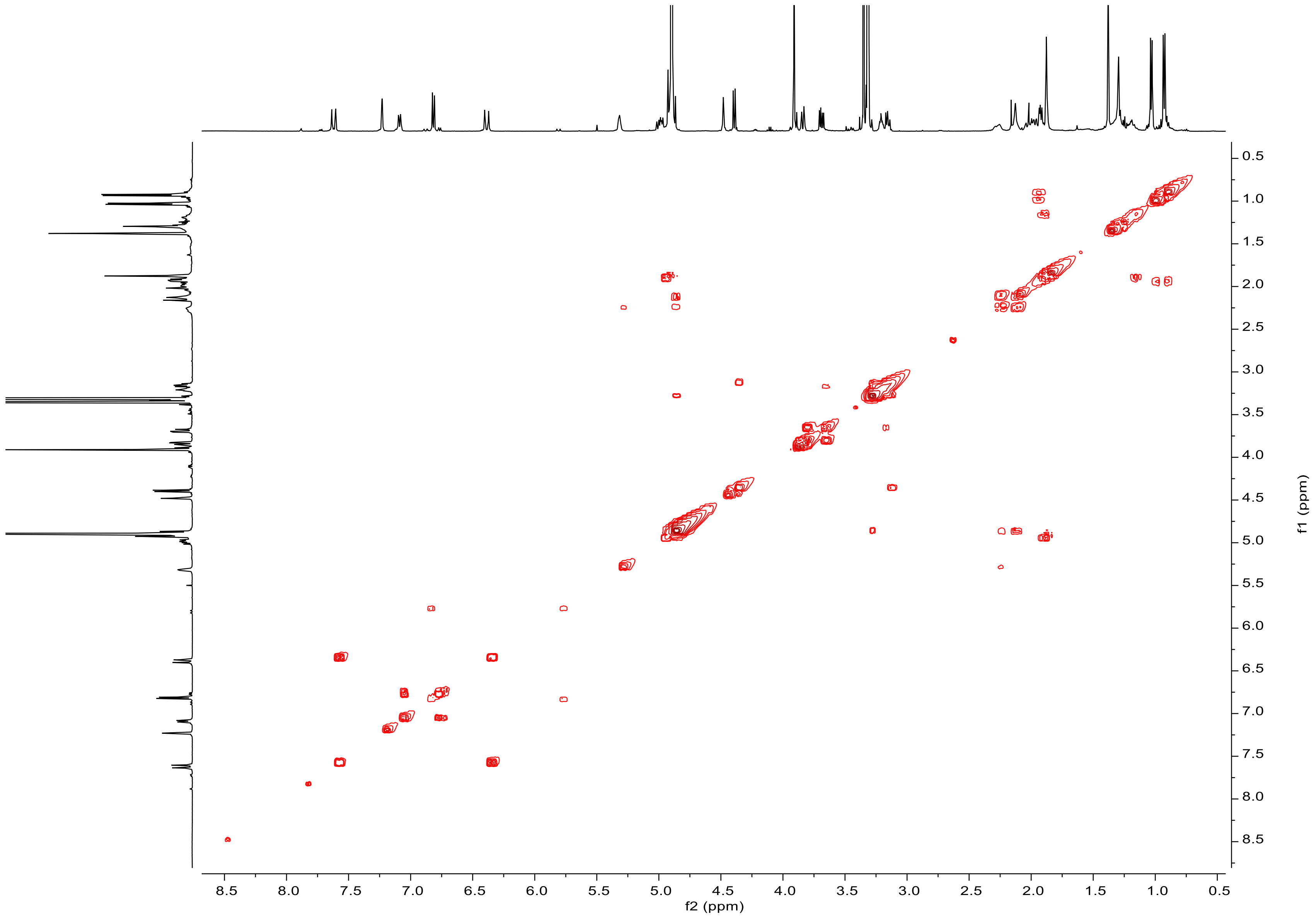

Figure S26. The HMBC spectrum of compound **7**

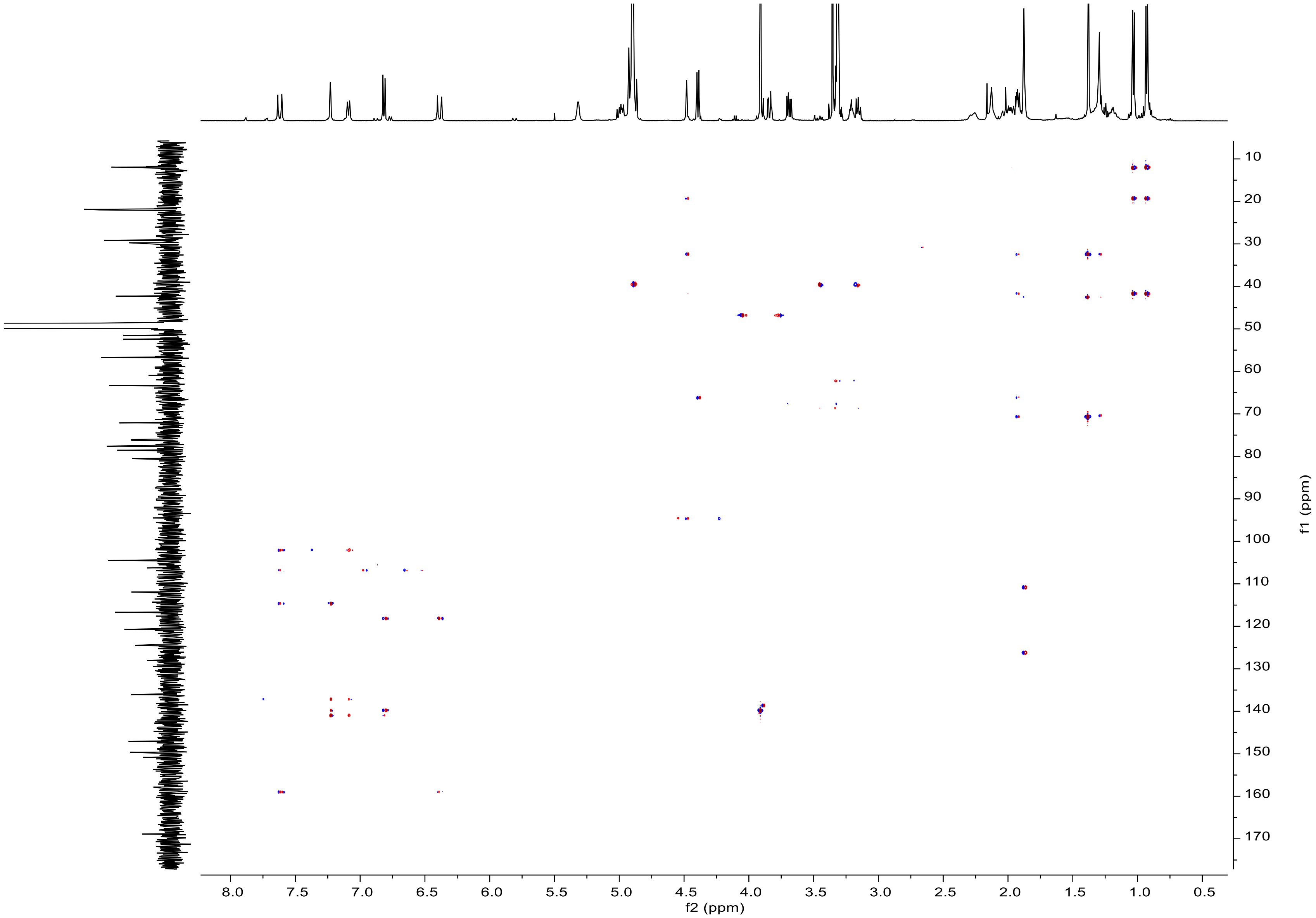

Figure S27. The NOESY spectrum of compound **7**

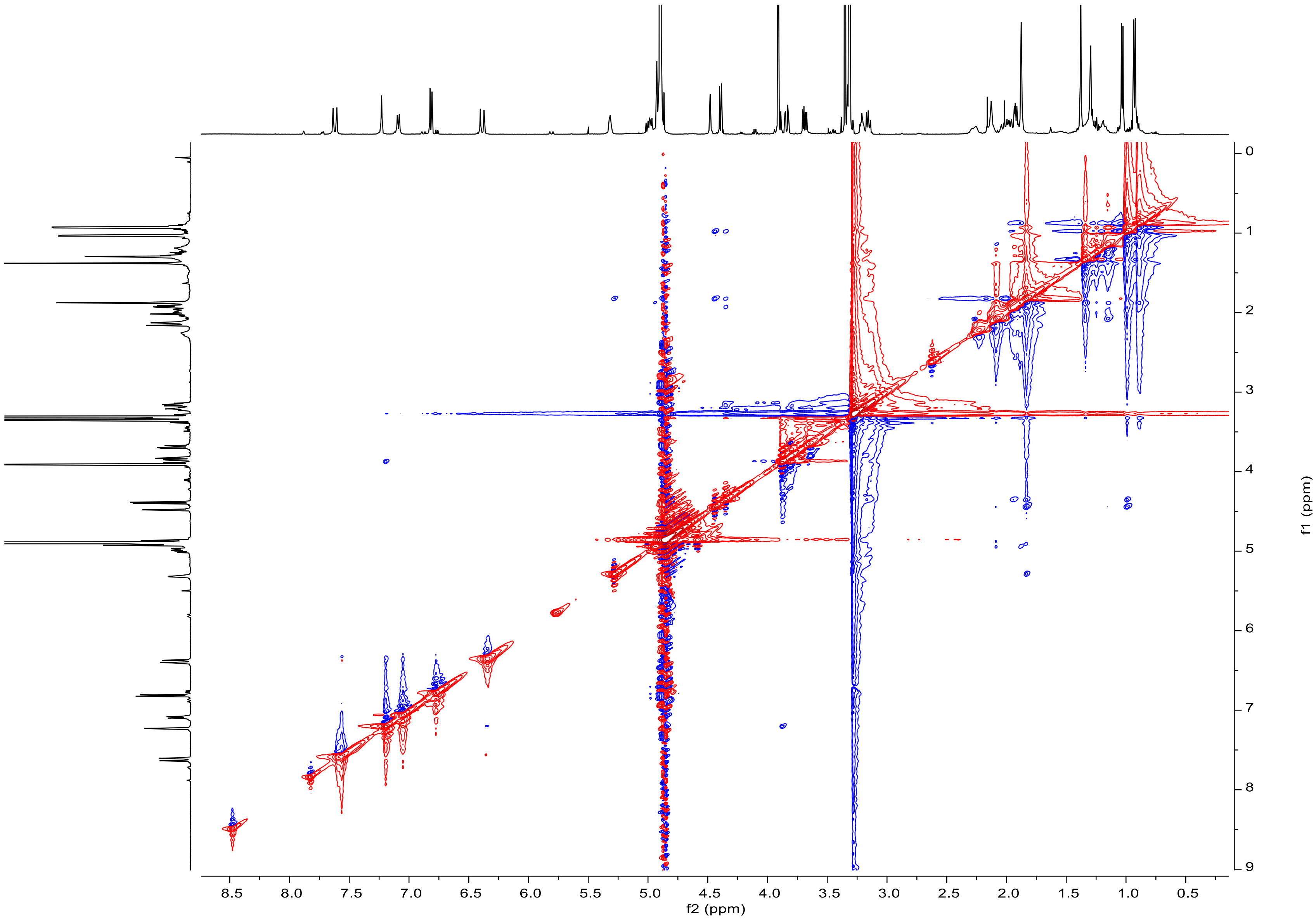

Supplement: Supplementary file 1 [file plants-09-01811-s001.pdf]
